# Heterologous Expression of the Unusual Terreazepine Biosynthetic Gene Cluster Reveals a Promising Approach for Identifying New Chemical Scaffolds

**DOI:** 10.1128/mBio.01691-20

**Published:** 2020-08-25

**Authors:** Lindsay K. Caesar, Matthew T. Robey, Michael Swyers, Md N. Islam, Rosa Ye, Purav P. Vagadia, Gary E. Schiltz, Paul M. Thomas, Chengcang C. Wu, Neil L. Kelleher, Nancy P. Keller, Jin Woo Bok

**Affiliations:** a Department of Chemistry, Northwestern University, Evanston, Illinois, USA; b Department of Molecular Biosciences, Northwestern University, Evanston, Illinois, USA; c Intact Genomics, Inc., St. Louis, Missouri, USA; d Center for Molecular Innovation and Drug Discovery, Northwestern University, Evanston, Illinois, USA; e Department of Pharmacology, Northwestern University, Chicago, Illinois, USA; f Robert H. Lurie Comprehensive Cancer Center, Feinberg School of Medicine, Northwestern University, Chicago, Illinois, USA; g Proteomics Center of Excellence, Northwestern University, Evanston, Illinois, USA; h Department of Medical Microbiology and Immunology, University of Wisconsin—Madison, Madison, Wisconsin, USA; i Department of Bacteriology, University of Wisconsin—Madison, Madison, Wisconsin, USA; University of British Columbia

**Keywords:** IDO, heterologous expression, natural products, *Aspergillus terreus*, *Aspergillus nidulans*, genome mining, *Aspergillus*, biosynthetic gene cluster, indoleamine 2,3-dioxygenase, kynurenine, NRPS

## Abstract

Advances in genome sequencing have revitalized natural product discovery efforts, revealing the untapped biosynthetic potential of fungi. While the volume of genomic data continues to expand, discovery efforts are slowed due to the time-consuming nature of experiments required to characterize new molecules. To direct efforts toward uncharacterized biosynthetic gene clusters most likely to encode novel chemical scaffolds, we took advantage of comparative metabolomics and heterologous gene expression using fungal artificial chromosomes (FACs). By linking mass spectral profiles with structural clues provided by FAC-encoded gene clusters, we targeted a compound originating from an unusual gene cluster containing an indoleamine 2,3-dioxygenase (IDO). With this approach, we isolate and characterize *R* and *S* forms of the new molecule terreazepine, which contains a novel chemical scaffold resulting from cyclization of the IDO-supplied kynurenine. The discovery of terreazepine illustrates that FAC-based approaches targeting unusual biosynthetic machinery provide a promising avenue forward for targeted discovery of novel scaffolds and their biosynthetic enzymes, and it also represents another example of a biosynthetic gene cluster “repurposing” a primary metabolic enzyme to diversify its secondary metabolite arsenal.

## Abstract (precis)

Here, we provide evidence that Aspergillus terreus encodes a biosynthetic gene cluster containing a repurposed indoleamine 2,3-dioxygenase (IDO) dedicated to secondary metabolite synthesis. The discovery of this neofunctionalized IDO not only enabled discovery of a new compound with an unusual chemical scaffold but also provided insight into the numerous strategies fungi employ for diversifying and protecting themselves against secondary metabolites. The observations in this study set the stage for further in-depth studies into the function of duplicated IDOs present in fungal biosynthetic gene clusters and presents a strategy for accessing the biosynthetic potential of gene clusters containing duplicated primary metabolic genes.

## INTRODUCTION

Fungal natural products (secondary metabolites) are an invaluable source of inspiration for pharmaceuticals that act against myriad conditions, including infectious diseases, cancer, and hyperlipidemia (1–4). Indeed, the antibiotics penicillin and cephalosporin, the cholesterol-lowering lovastatin, and the immunosuppressant cyclosporine are derived from fungi (5, 6), and the reservoir of novel scaffolds continues to grow each year (7). Although numerous drugs derived from fungi exist on the market today, genome sequencing has revealed that fungi possess the biosynthetic capacity to produce a far greater number of secondary metabolites than currently accessed (8). Recent studies spanning nearly 600 fungal genomes suggest that a mere 3% of molecules encoded by fungal biosynthetic gene clusters (BGCs) have been explored (8).

**FIG 1:**
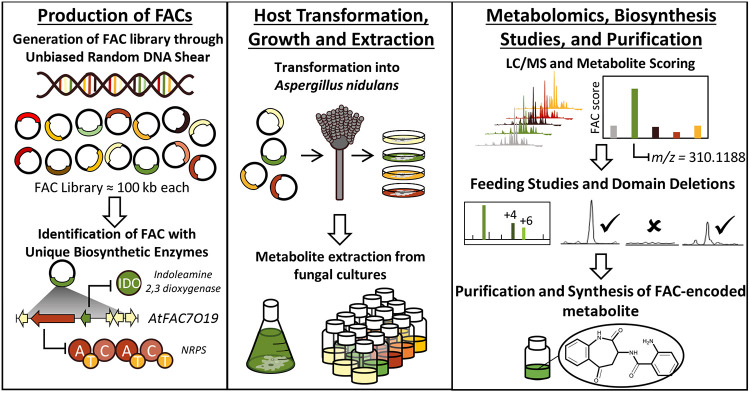
The fungal artificial chromosome (FAC)-metabolite scoring platform for discovering fungal secondary metabolites originating from unusual biosynthetic gene clusters.

To access this biosynthetic potential, an innovative discovery pipeline was recently developed to systematically annotate the biosynthetic abilities of fungi using comparative metabolomics and heterologous gene expression (9–12). With this platform, fungal genomic DNA fragments containing intact BGCs are inserted into fungal artificial chromosomes (FACs) and are used to transform a fungal host to discover new chemical scaffolds (10–12). This pipeline uses a metabolite scoring system to identify heterologously expressed metabolites from the thousands of signals originating from the host. By enabling facile linkage between secondary metabolites and their corresponding BGCs, the FAC-metabolite scoring pipeline facilitates prioritization of target compounds most likely to contain novel scaffolds. Using structural clues provided by BGC data, it is possible to target compounds originating from BGCs containing unusual biosynthetic machinery (Fig. 1).

Aromatic amino acids are fundamental for growth and development across phylogenetic kingdoms. Additionally, catabolism of aromatic amino acids leads to the production of nonproteinogenic amino acids, such as the tryptophan-derived kynurenine, which regulates inflammation and immune responses (13, 14). Kynurenine and its derivatives are biosynthetic intermediates of numerous secondary metabolites, including sibiromycin (15), mycemycin C (16), nidulanin A (17), nidulanin B and nidulanin D (18), daptomycin (19), and quinomycin peptide antibiotics (20). Recently, the malpikynines were discovered from Mortierella alpina as degradation products of the fungal surfactant malpinins, resulting from an oxidative conversion of tryptophan into kynurenine (21). Incorporation of kynurenine into secondary metabolites enables differential specificity toward enzyme receptors and targets (22). Daptomycin, for example, shows decreased antimicrobial efficacy when kynurenine is mutated to tryptophan (23, 24). One tactic for creating secondary metabolites with novel scaffolds is to recruit primary metabolic enzymes that modify common precursors into nonproteinogenic precursors into BGCs (20). For example, a tryptophan 2,3-dioxygenase (TDO) gene located adjacent to the daptomycin-producing nonribosomal peptide synthetase (NRPS) gene encodes an enzyme that supplies the kynurenine for daptomycin synthesis. This TDO diverges from related proteins in the same genus (29% sequence identity), suggesting it is a paralogous enzyme dedicated to secondary metabolite biosynthesis (19).

**FIG 2:**
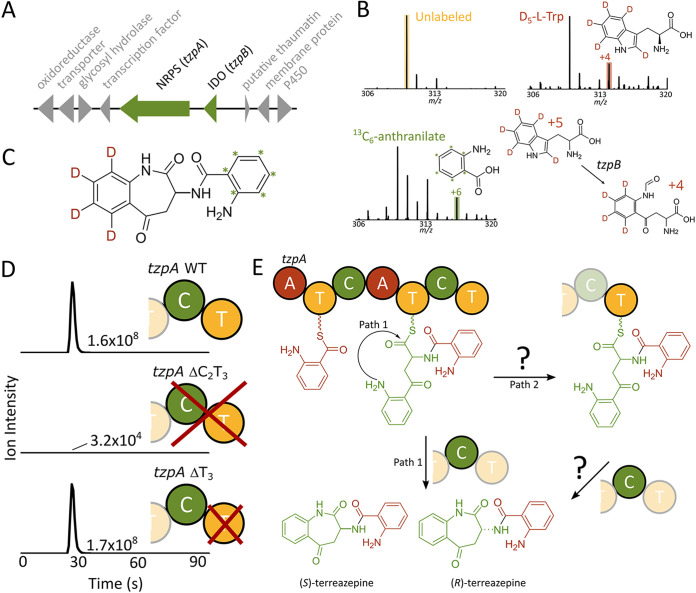
Proposed terreazepine biosynthetic pathway. (A) The terreazepine biosynthetic gene cluster. (B) Mass spectral shifts of terreazepine following feeding with l-tryptophan-D_5_ and [^13^C_6_]anthranilate. (C) Proposed incorporation of isotope-labeled precursors into terreazepine. (D) Selected ion chromatograms of terreazepine in *tzpA* domain deletion mutants. (E) Proposed NRPS assembly of terreazepine. It remains unclear whether the final cyclization event can occur from both T_2_ and T_3_ domains.

In a large-scale analysis of 56 FACs, an unknown metabolite from heterologous expression of a BGC from Aspergillus terreus ATCC 20542 (located on the FAC AtFAC7O19; Fig. 2A; also see Table S1 in the supplemental material) was identified with an *m/z* value of 310.1188 and a molecular formula of C_17_H_15_N_3_O_3_ (calculated [M+H]^+^ mass 310.1192, −0.97 ppm) (10). This compound was found in both the parent strain and the AtFAC7O19-transformed Aspergillus nidulans, but not in the empty vector control. Interestingly, the BGC encoding this metabolite contained an indoleamine 2,3-dioxygenase (IDO), which is involved in tryptophan degradation via kynurenine production (25). While most aspergilli contain three IDOs, A. terreus contains four (see Fig. S1 in the supplemental material). Given that gene duplication is often utilized as a strategy to “repurpose” genes for secondary metabolism, the presence of this fourth IDO suggested that it may serve to supply kynurenine for the formation of the unknown metabolite. Here, we employ the FAC-metabolite scoring strategy to identify the biosynthetic product of this unusual gene cluster and probe its biosynthesis.

10.1128/mBio.01691-20.2 FIG S1 (A) Phylogenetic tree of IDOs in a subset of aspergilli. *idoA*, *idoB*, and *idoC* homologs form distinct clades, as annotated according to reference sequences from A. fumigatus and A. oryzae. Interestingly, *tzpB* and other duplicated IDOs cluster together and share moderate sequence homology to both *idoA* and *idoB*. (B) Average IDO counts in aspergilli. Download FIG S1, PDF file, 0.4 MB. Copyright © 2020 Caesar et al. 2020 Caesar et al. This content is distributed under the terms of the Creative Commons Attribution 4.0 International license.

10.1128/mBio.01691-20.8 TABLE S1 (A) Annotated boundaries of AtFAC7O19 in comparison with the A. terreus NIH2624 reference genome. (B) Primers and oligonucleotides used to produce gene and NRPS domain deletions. Download Table S1, PDF file, 0.2 MB. Copyright © 2020 Caesar et al. 2020 Caesar et al. This content is distributed under the terms of the Creative Commons Attribution 4.0 International license.

## RESULTS

To determine the structure of the target compound, ∼1.5 mg of material was purified from FAC-transformed A. nidulans extracts and subjected to tandem mass spectrometry (MS^2^) analysis, ^1^H and ^13^C nuclear magnetic resonance (NMR) spectroscopy, and two-dimensional (2D) correlation approaches, including correlation spectroscopy (COSY), heteronuclear single quantum correlation (HSQC), and heteronuclear multiple bond correlation (HMBC) (see Table S2 and Fig. S2 and S3 in the supplemental material). Structural analysis revealed an unusual secondary metabolite backbone, a 3,4-dihydro-1*H*-1-benzazepine-2,5-dione, resulting from the unusual cyclization of kynurenine. The metabolite’s structure matches that of a previously synthesized kynurenine derivative, 2-amino-*N-*(2,3,4,5-tetrahydro-2,5-dioxo-1*H-*1-benzazepin-3-yl)benzamide (26). On the basis of its structure and the parent organism, it was given a common name of “terreazepine.” To determine the stereochemical configuration of terreazepine, *R* and *S* enantiomers were synthesized, each with an enantiomeric excess of ≥95% (Fig. S4). Each enantiomer and the purified natural compound were acylated to enable separation using supercritical fluid chromatography. Interestingly, natural terreazepine was found to be a 2:1 mixture of *S*-*R* enantiomers by comparing against synthetic standards separated by chiral chromatography (Fig. S4). During the course of this work, Li et al. reported the independent discovery of (*S*)-terreazepine (nanangelenin B) as an intermediate in the biosynthesis of the related compound nanangelenin A (27). Given that our discovery of terreazepine was completed prior to the publication by Li et al., the strategies outlined herein nonetheless provide promise for the discovery of novel chemical scaffolds.

10.1128/mBio.01691-20.3 FIG S2 MS^2^ fragmentation spectra for terreazepine, fragmented through HCD at a normalized collision energy of 25.0%. Download FIG S2, PDF file, 0.1 MB. Copyright © 2020 Caesar et al. 2020 Caesar et al. This content is distributed under the terms of the Creative Commons Attribution 4.0 International license.

10.1128/mBio.01691-20.4 FIG S3 (A) Overlapping ^1^H NMR spectra for natural (top) and synthetic (bottom) terreazepine in DMSO-*d_6_*. ^1^H signals are consistent between samples, indicating that the correct product was obtained through synthesis. (B) Overlapping ^13^C NMR spectra for natural (top) and synthetic (bottom) terreazepine in DMSO-*d_6_*. ^13^C signals are consistent between samples, indicating that the correct product was obtained through synthesis. (C) ^1^H NMR data for synthetic (*R*) -terreazepine (500 MHz, DMSO-*d_6_*). (D) ^13^C NMR data for synthetic (*R*)-terreazepine (125 MHz, DMSO-*d_6_*). (E) COSY data for synthetic (*R*)-terreazepine (500 MHz, DMSO-*d_6_*). (F) HSQC data for synthetic (*R*)-terreazepine (500 MHz, DMSO-*d_6_*). (G) HMBC data for synthetic (*R*)-terreazepine (500 MHz, DMSO-*d_6_*). (H) ^1^H NMR spectra for (*S*)-terreazepine in methanol-*d_4_* (500 MHz). (I) ^13^C NMR spectra for (*S*)-terreazepine in methanol-*d_4_* (125 MHz). Download FIG S3, PDF file, 0.9 MB. Copyright © 2020 Caesar et al. 2020 Caesar et al. This content is distributed under the terms of the Creative Commons Attribution 4.0 International license.

10.1128/mBio.01691-20.5 FIG S4 Supercritical fluid chromatography (SFC) results for the acylated terreazpine racemic mixture (A), acylated synthetic (*S*)-enantiomer (B), acylated synthetic (*R*)-enantiomer (C), and acylated natural terreazepine (D). Download FIG S4, PDF file, 0.3 MB. Copyright © 2020 Caesar et al. 2020 Caesar et al. This content is distributed under the terms of the Creative Commons Attribution 4.0 International license.

10.1128/mBio.01691-20.9 TABLE S2 NMR data for terreazepine in DMSO-*d_6_.* ^1^H, COSY, HMBC, and HSQC data were collected at 500 MHz, and ^13^C data were collected at 125 MHz. Overlapping assignments (indicated by asterisks) were determined using HSQC and HMBC data. Download Table S2, PDF file, 0.1 MB. Copyright © 2020 Caesar et al. 2020 Caesar et al. This content is distributed under the terms of the Creative Commons Attribution 4.0 International license.

To probe terreazepine’s biosynthesis, we grew A. terreus (ATCC 20542) using media containing isotopically labeled biosynthetic precursors. Labeling with [^13^C_6_]anthranilate resulted in an *m/z* shift of +6 Da (Fig. 2B), supporting incorporation of anthranilate into the molecule (Fig. 2C). Consistent with terreazepine’s chemical structure, labeling with l-Trp-D_5_ did not result in the expected shift of +5 in the mass spectrum, instead resulting in a mass shift of +4 (Fig. 2B). Given the existence of an IDO in the AtFAC7O19 BGC, these data provide support that tryptophan is converted into kynurenine prior to incorporation into terreazepine. For further confirmation of the IDO activity in terreazepine biosynthesis, a FAC deletion mutant was produced lacking the IDO *tzpB* by RedET-mediated recombineering (10). Mass spectral analysis of the FAC deletion mutant revealed no terreazepine production (Fig. S5).

10.1128/mBio.01691-20.6 FIG S5 Selected ion chromatograms of terreazepine in FAC control (top) and *tzpB* deletion mutants (bottom). The very low production of terreazepine in the deletant strain confirms the involvement of the IDO in terreazepine production. Download FIG S5, PDF file, 0.1 MB. Copyright © 2020 Caesar et al. 2020 Caesar et al. This content is distributed under the terms of the Creative Commons Attribution 4.0 International license.

Homology-based annotation of the FAC-encoded NRPS revealed a domain structure consisting of two adenylation (A), two condensation (C), and three thiolation (T) domains, giving the domain sequence A_1_-T_1_-C_1_-A_2_-T_2_-C_2_-T_3_. To investigate the function of the seemingly extraneous T_3_ domain, we constructed FAC truncation mutants either lacking the C_2_T_3_ domains (ΔC_2_T_3_) or only the T_3_ domain (ΔT_3_). These constructs were transformed into A. nidulans, and extracted metabolites were subjected to liquid chromatography-mass spectrometry (LC-MS) analysis. A very small amount of the target compound was detected in ΔC_2_T_3_ extracts (5,000-fold lower than control), suggesting that terreazepine formation can occur slowly without catalysis. We were unable to detect the presence of any offloaded intermediates. Interestingly, ΔT_3_ extracts contained terreazepine levels close to that of the intact NRPS (Fig. 2D). Given that analyses focused on endpoint abundance of terreazepine, it is possible that the T_3_ domain increases the catalytic efficiency of product formation. This is in contrast to recent findings by Li et al. in which NanA, the TzpA ortholog involved in nanangelenin A biosynthesis, requires the T_3_ domain for product formation (27). The reasons for these differences are unknown but may involve differences in protein sequence or other ancillary protein interactions.

Using heterologous expression, stable isotope feeding studies, and NRPS backbone deletions, we propose a biosynthetic scheme for terreazepine (Fig. 2E). In this scheme, *N*-formyl-kynurenine is formed through the catabolism of tryptophan by TzpB, an IDO. TzpB shares 41% sequence identity to Aspergillus fumigatus IdoA and 45% identity to IdoB, and only 26% identity to IdoC. Enzymatic studies using Aspergillus oryzae suggest that only *idoα* and *idoβ* (orthologs of *idoA* and *idoB*, respectively) encode enzymes that participate in tryptophan catabolism (28, 29). Because most aspergilli contain three IDOs, TzpB, a fourth IDO in the parent organism A. terreus, may no longer play a role in primary metabolism and instead represent a duplicated enzyme dedicated to terreazepine biosynthesis (Fig. S1). This is reminiscent of daptomycin biosynthesis in Streptomyces roseosporus, in which the TDO DptJ supplies kynurenine for daptomycin formation (19). The biosynthesis of terreazepine mirrors that of its relative nanangelenin A, where *tzpA* and *tzpB* orthologs in Aspergillus nanangensis (*nanA* and *nanC*) encode enzymes with near identical activity.

As the next step in the proposed biosynthesis, TzpA, a two-module NRPS, utilizes anthranilate and kynurenine to assemble terreazepine. The first adenylation domain (TzpA-A_1_) loads anthranilate onto the T_1_ domain, while TzpA-A_2_ loads kynurenine, generated through spontaneous nonenzymatic deformylation of the TzpB-supplied *N-*formyl-kynurenine. The substrate-binding residues of TzpA-A_1_ resemble those of other fungal adenylation domains that recognize anthranilate (Table S3). TzpA-A_2_, responsible for incorporating kynurenine, has a new pocket code quite dissimilar from other kynurenine-binding A-domains (Table S3). However, this disparity may be attributable to evolutionary distance between source organisms and the unstudied nature of kynurenine incorporation into fungal secondary metabolites. Given that the isolated terreazepine was a 2:1 mixture of *S*-*R* enantiomers, we can speculate that TzpA-A_2_ accepts both d- and l-kynurenine. The peptide bond formation between the tethered amino acids is catalyzed by the first condensation domain, TzpA-C_1_, between anthranilate’s carbonyl carbon and kynurenine’s aliphatic primary amine. The second C domain (TzpA-C_2_) catalyzes the final cyclization event between the aromatic amine of kynurenine and the tethered carbonyl carbon, yielding the final terreazepine product.

10.1128/mBio.01691-20.10 TABLE S3 Adenylation domain substrate predictions for TzpA, a nonribosomal peptide synthetase and C_2_, T_2_, and T_3_ domain active site sequence alignments. (A) TzpA-A_1_ substrate-binding residues bear similarity to many additional anthranilate-activating adenylation domains. Additionally, adenylation domains from *A. thermomutatus* (RHZ670305-A_1_) and A. lentulus (GATaQ05471-A_1_) have an identical A-domain sequence to that of TzpA-A_1_, suggesting they also bind anthranilate. (B) TzpA-A_2_ possesses a specificity sequence that is disparate from known kynurenine-binding A domains. It does, however, bear resemblance to the A_2_ domains from the orphan NRPSs RHZ670305-A_2_ and GAQ05471-A_2_ and may represent a new type of kynurenine-activating adenylation domain. (C) The C_2_ domain of TzpA does possess the catalytic histidine purported to be required for activity (J. A. Baccile, H. H. Le, B. T. Pfannenstiel, J. W. Bok, et al., Angew Chem Int 58:14589−14593, 2019, https://doi.org/10.1002/anie.201909052), although the remainder of its sequence diverges from other C_2_ domains part of NRPSs with the ATCATCT domain architecture such as GliP and HasD. (D) The T_2_ and T_3_ domains of TzpA both appear functional compared to GliP T domains and GrsA T domains with known functionality (G. L. Challis, J. Ravel, C. A. Townsend, Chem Biol 7:211–224, 2000, https://doi.org/10.1016/s1074-5521(00)00091-0) given their sequence similarity and the presence of a conserved serine in the sequence. Residues are colored according to the Taylor coloring scheme (W. R. Taylor, Protein Eng 10:743–746, 1997, https://doi.org/10.1093/protein/10.7.743). Download Table S3, PDF file, 0.3 MB. Copyright © 2020 Caesar et al. 2020 Caesar et al. This content is distributed under the terms of the Creative Commons Attribution 4.0 International license.

While the role of the terminal TzpA-T_3_ domain remains uncertain, we can glean insight by looking at related NRPSs. For example, the unusual NRPS domain structure of TzpA mirrors that of GliP, the NRPS involved in gliotoxin biosynthesis (30, 31). When studied *in vitro*, GliP mutants show behavior mirroring that of TzpA deletants: truncated GliP ΔT_3_ mutants retain dipeptide synthetase activity, while ΔC_2_T_3_ mutants show reduced activity (30, 31). However, *in vivo*, GliP ΔT_3_ loses activity, suggesting that the *in vivo* pathway involves transfer of the dipeptidyl-S intermediate from T_2_ to T_3_ (30). In light of these two possible pathways of cyclization from T_2_ and T_3_, as well as a slow reported rate of approximately 1 per h, it has been suggested that T_3_ facilitates interaction with downstream tailoring enzymes (30, 31). Given that terreazepine biosynthesis does not appear to require the activity of downstream tailoring enzymes, both cyclization pathways may exist. Like the T domains of GliP, TzpA-T_2_ and T_3_ possess the predicted active site residue (S1937 and S2473, respectively), suggesting that they are both functional (Table S3). Similarly, TzpA-C_2_ possesses the purported catalytic histidine at position H2137. However, the adjacent residue sequence diverges from the conserved SHXXXDXX(S/T) sequence shared by diketopiperazine-forming NRPSs such as GliP and HasD (30), and slightly from the SHXXXD sequence of NanA (27), suggesting it may have different cyclization requirements (Table S3). More studies will be required to elucidate the nuanced functions of terminal T domains in natural product biosynthesis.

**FIG 3:**
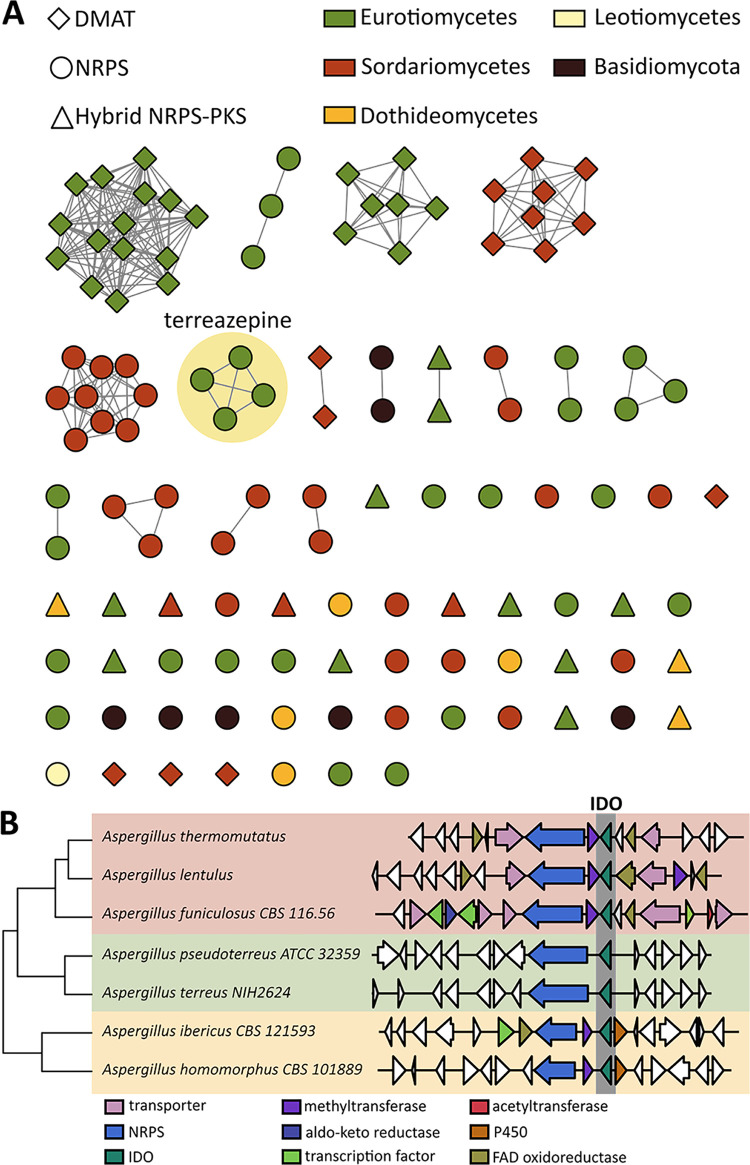
Diversity of indoleamine 2,3 diooxygenase (IDO)-containing BGCs across fungi. (A) Gene cluster families containing IDOs. (B) Distribution of selected IDO-containing biosynthetic gene clusters across diverse aspergilli. DMAT, dimethylallyl tryptophan synthase; PKS, polyketide synthase; FAD, flavin adenine dinucleotide.

The discovery of terreazepine and its BGC revealed that fungal IDOs can play a role in secondary metabolite biosynthesis and that kynurenine incorporation into secondary metabolites can yield novel chemical scaffolds. This suggests that targeted efforts to characterize fungal BGCs containing IDOs may facilitate the discovery of completely new molecules with unique chemical scaffolds and their derivatives. To explore this possibility, we searched sequences of 1,037 fungal genomes from GenBank and the Joint Genome Institute and located BGCs containing IDOs. Of the ∼38,000 BGCs contained within these genomes, 118 contain an IDO. We then grouped IDO-containing BGCs into gene cluster families (GCFs) based on sequence identity and the fraction of protein domains shared between BGC pairs, anticipating that a single GCF groups BGCs that produce similar metabolites. Of the 118 IDO-containing BGCs, 68 were sorted into 16 GCFs. The remaining 50 BGCs represent singletons that had no similar BGC pairs (Fig. 3A).

Several interesting insights can be gleaned from this analysis. First, many BGCs originate from phylogenetically diverse aspergilli, an NRPS-containing subset of which are illustrated in Fig. 3B. BGCs from two *Aspergillus* GCFs in particular were identified as putative terreazepine clusters. The first GCF includes the terreazepine BGC itself, which exists in Aspergillus terreus and A. pseudoterreus. The second GCF contains BGCs from Aspergillus thermomutatus, A. funiculosus, and A. lentulus. The NRPSs in this GCF follow the same unusual domain sequence of ATCATCT (with the exception of A. lentulus which lacks the terminal T domain). Adenylation domain specificity codes bear remarkable similarity to those of TzpA-A_1_ and TzpA-A_2_ (Table S3), suggesting that these NRPSs biosynthesize terreazepine. Unlike the terreazepine BGC, however, the BGCs in this family contain several tailoring enzymes expected to diversify the terreazepine scaffold, raising the possibility that the shared NRPS T_3_ facilitates interaction with downstream enzymes in these pathways. The tailoring enzymes present in these BGCs differ from those present in the nanangelenin A cluster in A. nanangensis, indicating that a variety of terreazepine/nanangelenin analogs may exist (27). Moreover, IDO-containing BGCs from Aspergillus ibericus and A. homomorphus may encode yet undiscovered dipeptide scaffolds containing kynurenine (Fig. 3B). Interestingly, the IDOs contained in these three GCFs represent a distinct clade of duplicated IDOs with moderate sequence homology (∼40%) to both A. fumigatus IdoA and IdoB (Fig. S1).

Perhaps even more remarkable is the degree to which IDO-containing BGCs span the kingdom of fungi, encompassing five taxonomic classes and two phyla (Fig. S6). Particularly interesting is the presence of several NRPS-containing BGCs originating from Basidiomycetes, given the rare and unstudied nature of NRPSs in this phylum (32). Taken together, these results reveal the rich biosynthetic potential of IDO-containing BGCs that has only just begun to be explored.

10.1128/mBio.01691-20.7 FIG S6 IDO-containing biosynthetic gene clusters in fungi. These gene clusters encompass a wide range of phylogenetically diverse fungi with diverse backbone gene domain sequences. Download FIG S6, PDF file, 0.7 MB. Copyright © 2020 Caesar et al. 2020 Caesar et al. This content is distributed under the terms of the Creative Commons Attribution 4.0 International license.

## DISCUSSION

The work described herein has elucidated a clear biosynthetic role for two FAC-encoded enzymes, TzpA, an NRPS, and TzpB, an IDO, in the biosynthesis of terreazepine. A closer look at the genes surrounding *tzpA* and *tzpB*, however, reveals additional genes that could play a role in biosynthesis. Indeed, adjacent genes include an alcohol dehydrogenase (ATEG_07354), a transcription factor (ATEG_07357), and a P450 (ATEG_07362) (Fig. 2A; see Table S1 in the supplemental material). While we were unable to determine whether these additional genes encode regulatory elements or biosynthetic players involved in terreazepine formation, a few possibilities warrant discussion.

First, it is possible that the tailoring enzymes present in the gene cluster (i.e., the P450 and the alcohol dehydrogenase) remain silent and that the gene cluster itself is only partially expressed. Given that only (*S*)-terreazepine is formed as an intermediate in nanangelenin A production in A. nanangensis (27), a second possibility is that these tailoring enzymes play a role in the epimerization of (*S*)-terreazpine to (*R*)-terreazepine. This epimerization could occur through a two-step mechanism similar to that of the conversion of (*S*)-reticuline to (*R*)-reticuline in Saccharomyces cerevisiae (33). Should this be the case, it would follow that TzpA produces (*S*)-terreazepine which is then oxidized from an amine to an enamine by the P450. This oxidized product could then be reduced by the alcohol dehydrogenase/oxidoreductase to form the *S*-*R* mixture that we observed. Of course, the activities of these enzymes are at this point only speculative, and future studies will be required to confirm what, if any, roles these enzymes play in terreazepine biosynthesis.

**FIG 4:**
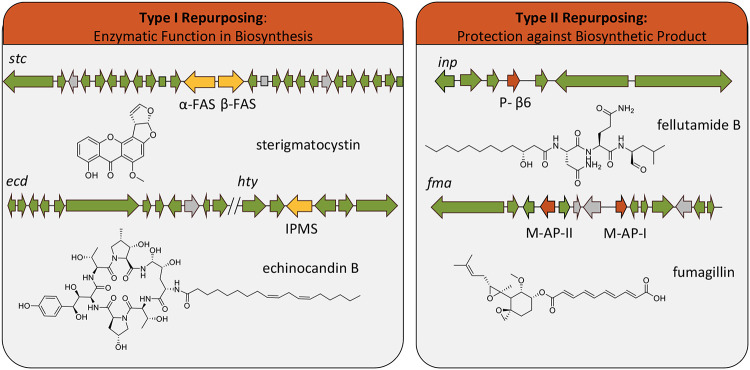
Type I and type II primary metabolism gene repurposing strategies. Green arrows represent biosynthetic genes, including backbone genes, tailoring genes, and their regulatory elements. Gray arrows represent hypothetical proteins or genes unrelated to biosynthesis. Yellow arrows found in sterigmatocystin (*stc*) and echinocandin B (*ecd* and *hty*) biosynthetic gene clusters represent examples of type I repurposing of primary metabolism genes, and red arrows in fellutamide B (*inp*) and fumagillin (*fma*) gene clusters represent examples of type II repurposed primary metabolism genes. FAS, fatty acid synthase; IPMS, isopropylmalate synthase; P-β6, proteasome β6 subunit; M-AP, methionine aminopeptidase.

The discovery of terreazepine provides another example of how fungi repurpose primary metabolism genes for secondary metabolism. On the basis of this and other examples, we propose two major strategies fungi employ for such repurposing: type I repurposing into biosynthetic enzymes and type II repurposing into resistance genes (Fig. 4). One of the earliest discoveries of type I repurposing is that of the important fungal toxin sterigmatocystin. Evaluation of the sterigmatocystin biosynthetic pathway revealed the presence of two fatty acid synthase (FAS) genes, *stcJ* and *stcK* located within the sterigmatocystin gene cluster. Indeed, disruption of these genes in A. nidulans resulted in strains that did not produce sterigmatocystin but were morphologically identical to wild-type strains (34). Another important example of type I repurposing is the duplicated isopropyl-malate synthase (IPMS) involved in echinocandin biosynthesis in Emericella rugulosa. Similar to the provision of kynurenine by TzpB, this duplicated IPMS serves to provide the nonproteinogenic amino acid homotyrosine for incorporation into echinocandin B (Fig. 4) (35).

In addition to repurposing duplicated primary metabolism genes to have a biosynthetic role, fungi also utilize duplicated genes from primary metabolism as a form of self-resistance (36, 37). This type II repurposing represents a particularly attractive avenue for drug discovery, as the duplicated gene will often provide insight into the mechanism of action of the encoded secondary metabolite. Several examples of such type II repurposing have been discovered by targeting clusters with duplicate resistance targets. The proteasome inhibitor fellutamide B, for example, was discovered due to the presence of a duplicated proteasome subunit within its BGC (38). Similarly, the BGC encoding the methionine aminopeptidase inhibitor fumagillin contains both type I and type II methionine aminopeptidase genes in the gene cluster (Fig. 4) (39). While it is likely that many of the IDOs contained within the BGCs depicted in Fig. 3 and Fig. S6 represent type I biosynthetic enzymes that provide kynurenine for secondary metabolite synthesis, it is also possible that they represent type II duplicated gene targets that serve to protect the producing organism against the biosynthetic product. Indeed, when we began this research, we suspected that terreazepine might possess IDO inhibitory activity and show promise as an anticancer agent (40). When tested against A. fumigatus IDO mutants, however, no growth inhibitory activity was observed (data not shown). Future studies aimed to elucidate the biosynthetic products of additional IDO-containing BGCs in fungi offer exciting opportunities not only to discover new molecular scaffolds but also to identify anticancer metabolites with known mechanisms of action.

In sum, this work has shown the feasibility of using FACs to discover secondary metabolites from unusual BGCs. With this approach, we identified, reconstructed, cloned, and heterologously expressed an unusual BGC encoding the novel metabolite terreazepine. Thus far, IDO involvement in secondary metabolite biosynthesis in fungi has only just begun to be explored, suggesting that future efforts targeting BGCs including IDOs and other unusual biosynthetic players may prove fruitful. By mapping BGC relatedness across the fungal kingdom, we also uncover evidence of numerous IDO-containing BGCs in phylogenetically diverse fungi that may encode metabolites with novel chemical scaffolds or IDO inhibitory activity. This approach shows promise for focusing natural product discovery efforts toward uncharted biosynthetic space, yielding a diverse array of novel molecular scaffolds.

## MATERIALS AND METHODS

### FAC Generation, transformation, growth, and extraction.

AtFAC7O19 was produced in a prior study through the unbiased random shear of Aspergillus terreus ATCC 20542 genomic DNA (10). Using previously described methods, targeted metabolomics and FAC metabolite scoring identified a target compound with *m/z* 310.1188 that was likely to be produced from this FAC (10). AtFAC7O19 was then maintained in Escherichia coli strain DH10B and used to transform the Red/ET-inducible E. coli strain SW012 prior to DNA preparation and transformation of A. nidulans, as described in a previous study (11). Following transformation of A. nidulans, growth and extraction of FAC-containing and control strains were completed as previously reported (10).

### LC-MS analysis.

Dried metabolite extracts were resuspended in methanol to a concentration of 10 mg/ml. Samples were sonicated for 10 min and then centrifuged for 10 min at 21,000 × *g*. Samples were analyzed by LC-MS using a Thermo Q Exactive mass spectrometer with an inline Agilent 1290 ultrahigh performance liquid chromatograph (UHPLC) with a Phenomenex Kinetix 1.3-μm-particle-size column with dimensions 2 × 100 mm. Five percent acetonitrile in water with 0.01% formic acid was used as buffer A, and 100% acetonitrile with 0.01% formic acid was used as buffer B. An 8 min, 0 to 100% buffer B, gradient was used. The MS heated electrospray ionization (HESI) source was set at 275°C, with sheath gas set at 60 arbitrary units and spray voltage set at 3.5 kV. Data were collected in data-dependent mode, with the top five ions from each scan selected for higher-energy collisional dissociation (HCD) MS^2^ fragmentation analysis with a normalized collision energy of 25.0%. A resolution of 17,500 was used for both MS^1^ and MS^2^.

### Stable isotope labeling studies.

Isotopically labeled media were prepared by adding 1 mM (each) [^13^C_6_]anthranilate or l-tryptophan-D_5_ to plates of minimal medium containing glucose. The plates were inoculated with concentrated spore stocks of A. terreus and incubated at 30°C for 5 days. Metabolites were extracted from the plates using Oasis hydrophilic lipophilic balance solid phase extraction cartridges, with elution using 100% methanol. Extracts were dried *in vacuo* and analyzed by LC-MS as described above.

### Sequence analysis of AtFAC7019.

To compare the AtFAC7019 DNA sequence to that of the A. terreus reference strain NIH 2624, a barcoded Illumina next-generation sequencing library of AtFAC7O19 was generated using a True-Seq kit and pooled for sequencing with other samples at the University of Wisconsin–Madison Biotechnology Center DNA Sequencing Facility. A >1,000× coverage of the AtFAC7O19 DNA sequence was achieved. A single contig (114.911 kb, completed and finished FAC clone sequence) was obtained using the DNAStar next-generation sequence assembly program. The entire FAC sequence was confirmed by FAC end sequences, PmeI and NotI digestion, and contour-clamped homogenous-electric field gel electrophoresis. From this sequencing, we identified 35,769 kb of extra sequence toward the telomere in AtFAC7O19 that was not present in the reference genome (NIH 2624). However, the terreazepine gene cluster is well conserved in both strains. The AtFAC7O19 sequence is available through GenBank under accession number MT376588. Genes have been annotated in Table S1A in the supplemental material.

### Gene cluster editing and production of gene and domain deletants.

Gene and domain deletions were made with a modified method as previously described for whole-genome deletions (10, 11). Briefly, we used a GalK-gpdA-p gene cassette for overexpression of the transcription factor (TF) gene (ATEG_07357), a kanamycin gene cassette for deleting both ATEG_07358 (NRPS, *tzpA*) and ATEG_07359 (IDO, *tzpB*), and a GalK-apramycin (Ap) gene cassette for domain deletions as previously reported for whole-gene deletions. For example, to delete the T_3_ or C_2_T_3_ domains of *tzpA*, a pair of primers for the GalK and apramycin resistance gene cassette (24 bases forward and 23 bases reverse shown in Table S1B as lowercase letters) with an additional 50 bp (uppercase letters shown in Table S1B) of either T_3_ or C_2_T_3_ flanking to generate the GalK-Ap PCR amplicons. Gel-purified PCR amplicons were used to transform RedET-inducible E. coli strain SW102. Recombineered FAC colonies were then selected on LB medium with apramycin, and second-step recombineering transformation using either the AtFAC7O19-58ΔT or AtFAC7O19-58-ΔCT oligonucleotide enabled excision of the GalK-Ap gene cassette. All FAC recombinants were confirmed by PCR, restriction digestion, pulsed-field gel electrophoresis, and sequencing as needed. The primers for deletions are listed in Table S1B.

### Purification and structural analysis of terreazepine.

AtFAC7O19-transformed A. nidulans were grown on 576 plates and extracted as described, yielding 400 mg of dried extract. The crude extract was subjected to normal-stage flash chromatography using a CombiFlash NextGen 300 system (Teledyne-ISCO) and examined using UV absorbance at 254 and 280 nm. The extract was separated using a silica 12-g gold column (Teledyne-ISCO) at a flow rate of 30 ml/min with a 50-min hexane/CHCl_3_/methanol (MeOH) gradient. Of the 12 resulting simplified fractions, fraction 6 contained the highest concentration of the target metabolite and was subjected to reversed-phase separation using an Agilent 1200 HPLC and analyzed using OpenLAB CDS Chemstation software (version number 1.8.1, Agilent Technologies). Fraction 6 (56.9 mg) was separated on a Kinetix C_18_ semipreparatory column (5 μm; 100 Å; 250 × 10.0 mm; Phenomenex) at a flow rate of 2.5 ml/min. Approximately 200 μl of a 10-mg/ml solution was injected per run, and subsequent fractions were pooled together for analysis. The 45-min run began at 20:80 CH_3_CN-H_2_O and was increased to 40:60 over 20 min. The gradient was increased to 100% CH_3_CN over the next 20 min and held at 100% for the remainder of the run. Terreazepine eluted from 16 to 17.5 min (1.5 mg, 90% purity). All solvents used for chromatographic separation were acquired through Sigma-Aldrich and were HPLC-grade. MS^2^ spectra for terreazepine are shown in Fig. S2.

Nuclear magnetic resonance (NMR) spectra for the target compound, including ^1^H, ^13^C, correlation spectroscopy (COSY), heteronuclear multiple bond correlation (HMBC), and heteronuclear single quantum correlation (HSQC) spectra, were obtained using a Bruker Avance III 500-MHz system equipped with a DCH CryoProbe at 298.2 K. NMR spectra from the natural compound match those of the synthesized compound (see “Total synthesis and stereochemical analysis of terreazepine” below and Fig. S3). NMR spectra of the synthetic compound in DMSO-*d_6_* are the most complete and were used for full structural characterization (Table S2 and Fig. S3A to S3G). ^1^H and ^13^C NMR spectra are also provided for the synthetic compound in methanol-*d_4_* (Fig. S3H and S3I).

^1^H NMR (500 MHz, DMSO-*d_6_*) δ 3.02 (dd, J = 18.7, 2.6 Hz, 1H), 3.24 (dd, J = 18.7, 13.3 Hz, 1H), 4.99 (ddd, J = 13.2, 7.4 Hz, 2.4, 1H), 6.38 (br s, 2H), 6.54 (t, J = 7.9 Hz, 1H), 6.69 (d, J = 8.1 Hz, 1H), 7.17 (m, 1H), 7.19 (d, J = 8.0 Hz, 1H), 7.27 (t, J = 7.6 Hz, 1H), 7.58 (d, J = 7.6 Hz, 1H), 7.61 (td, J = 7.2, 1.4 Hz, 1H), 7.76 (dd, J = 7.9, 1.7 Hz, 1H), 8.42 (d, J = 7.4 Hz, 1H), 10.38 (s, 1H). ^13^C NMR (125 MHz, DMSO-*d_6_*) δ 45.75, 46.14, 113.87, 114.59, 116.36, 122.23, 124.36, 128.47 (2C), 130.12, 132.13, 134.25, 137.72, 149.70, 168.63, 171.25, 197.76.

^1^H NMR (500 MHz, methanol-*d*_4_) δ 7.89 (dd, *J *= 7.9, 1.6 Hz, 1H), 7.62 (td, *J *= 7.8, 1.7 Hz, 1H), 7.57 (dd, *J *= 7.9, 1.5 Hz, 1H), 7.32 (td, *J *= 7.5, 1.1 Hz, 1H), 7.22 (ddd, *J *= 8.5, 7.1, 1.6 Hz, 1H), 7.18 (dd, *J *= 8.1, 1.1 Hz, 1H), 6.76 (dd, *J *= 8.2, 1.1 Hz, 1H), 6.66 (ddd, *J *= 8.1, 7.2, 1.1 Hz, 1H), 5.18 (dd, *J *= 12.4, 3.5 Hz, 1H), 3.26 (dd, *J *= 18.8, 12.3 Hz, 1H), 3.19 (dd, *J *= 18.8, 3.5 Hz, 1H). ^13^C NMR (126 MHz, methanol-*d_4_*) δ 197.84, 171.93, 169.80, 149.11, 137.29, 134.12, 132.13, 130.11, 129.00, 127.83, 124.76, 122.14, 116.75, 115.87, 115.24, 46.50, 46.24.

The ^1^H NMR spectrum (Fig. S3C) enabled detection of two CH_2_ protons (δ 3.02 and 3.24), one CH proton (δ 4.99), two amide protons (δ 8.42 and 10.38), two NH_2_ protons (δ 6.38), and eight aromatic protons (δ 6.54–7.76). This is consistent with the proposed molecular formula of C_17_H_15_N_3_O_3_. The presence of eight proton signals in the aromatic region is consistent with the presence of two di-substituted benzene rings, while the three protons in the aliphatic region are consistent with the CH and CH_2_ species located in the seven-membered azepane-2,5-dione substructure. The downfield shift at H-11 (δ 4.99) is congruent with its adjacency to an amide nitrogen at position 12. The amide proton shift at δ 10.38 was readily assigned to the proton at position 2 given its adjacency to the aromatic ring system, while the remaining amide proton at H-12 was assigned to the δ 8.42 shift.

The ^13^C spectrum (Fig. S3D) contains only 16 signals, but one signal (δ 128.47) corresponds to two carbons, further supporting the proposed molecular formula. Three of the signals corresponded to carbonyls, including two amides and one ketone (δ 168.63, 171.25, and 197.76, respectively), one was a CH_2_ (δ 45.75), one was an N-CH (δ 46.14), and 12 were aromatic CH or quaternary signals (8 CHs and 4 quaternaries, δ 113.87–149.70). 2D experiments were used to correlate aromatic ^1^H and ^13^C bonds, as well as to determine the connectivity of the molecule.

The HSQC experiment enabled linkage of protons to their corresponding carbons (Fig. S3F), while the COSY and HMBC spectra helped to build connections through the molecule. Three distinct portions of the molecule are evident using COSY correlations linking protons H-4 through H-7, protons H-10 through H-12, and protons H-15 through H-18 (Fig. S3E). The remainder of the molecule could be pieced together using HMBC correlations (Fig. S3G). We determined that the site of cyclization was at position 1 due to the correlation between the amide proton at H-2 and the carbonyl carbon C-1.

### Total synthesis and stereochemical analysis of terreazepine.

Detailed descriptions and schemes for the total synthesis of (*R*)- and (S)-terreazepines as well as methods for the determination of their stereochemical configuration are provided in Text S1 in the supplemental material.

10.1128/mBio.01691-20.1 TEXT S1 Supplemental methods. Detailed methods for the syntheses and supercritical fluid chromatography of (*R*)- and (*S*)-terreazepine. Download Text S1, DOCX file, 0.1 MB. Copyright © 2020 Caesar et al. 2020 Caesar et al. This content is distributed under the terms of the Creative Commons Attribution 4.0 International license.

### Bioinformatics analysis.

**(i) Creation of gene cluster family networks.** We downloaded a data set of 1,037 fungal genomes from GenBank and the Joint Genome Institute Genome Portal, excluding Saccharomycotina as this subphylum contains a large number of genomes with very few gene clusters. Biosynthetic gene clusters were detected using the command-line version of antiSMASH 4 with default parameters (41). For creation of gene cluster families, we used an approach similar to those mentioned (42, 43). Protein domains were detected using the HMMER suite hmmsearch, resulting in protein domain arrays for each gene cluster. For each pair of gene clusters, the median sequence identity of backbone enzyme domains and the Jaccard similarity of protein domain sets was calculated. These two similarity metrics were combined for a single similarity score:

$$\text{similarity score}=\sqrt{(0.8\times \text{sequence identity)}+(0.2\times \text{Jaccard similarity)}}$$

A final clustering step was performed using DBSCAN clustering with an epsilon value of 0.55 (corresponding to a similarity score of 0.45). This process thus resulted in a network of gene clusters, where subgraphs correspond to gene cluster families.

All gene cluster family dendrograms (Fig. 3 and Fig. S6) were created using unweighted-pair group method using average linkages (UPGMA) clustering based on this same cluster similarity score.

**(ii) Sequence alignments.** All sequence alignments were performed using MUSCLE with default parameters in MEGA. For the data in Table S3, adenylation domain sequences from IDO-containing gene clusters were aligned together with known adenylation domain sequences from MIBiG. Putative substrate-binding residues were extracted using gramicidin S synthetase as a reference.

**(iii) Phylogenetic analyses.** A maximum likelihood phylogenetic tree was constructed using default parameters in MEGAX (44) to produce a tree containing IDOs in 15 *Aspergillus* species. The tree was constructed using 500 bootstrap iterations.

**(iv) Determining IDO counts per genome.** All *Aspergillus* genomes included in this study were analyzed using the HMMER suite tool *hmmsearch*, using the IDO Pfam model (PF01231) as a query model, a minimum subject sequence length of 350 residues, and a maximum E value of 1E−30.

### Data availability.

The AtFAC7O19 sequence is available through GenBank under accession number MT376588. Experimental details (Text S1) and other supporting data, including Fig. S1 to S6 (phylogenetic trees, structural elucidation data, stereochemical evaluation data, and expanded details of IDO-containing fungal gene clusters), and Tables S1 to S3 (genome sequence alignments and primers, NMR summary, adenylation domain specificity codes, and C and T domain alignments) are included in the supplemental material.
