# Supplementary material for: Heterologous Expression of the Unusual Terreazepine Biosynthetic Gene Cluster Reveals a Promising Approach for Identifying New Chemical Scaffolds
Source: mBio. 2020 Aug 25;11(4):e01691-20. doi: 10.1128/mBio.01691-20 (PMC7448278; doi:10.1128/mBio.01691-20)
Supplement: FIG S1 [file mBio.01691-20-sf001.pdf]

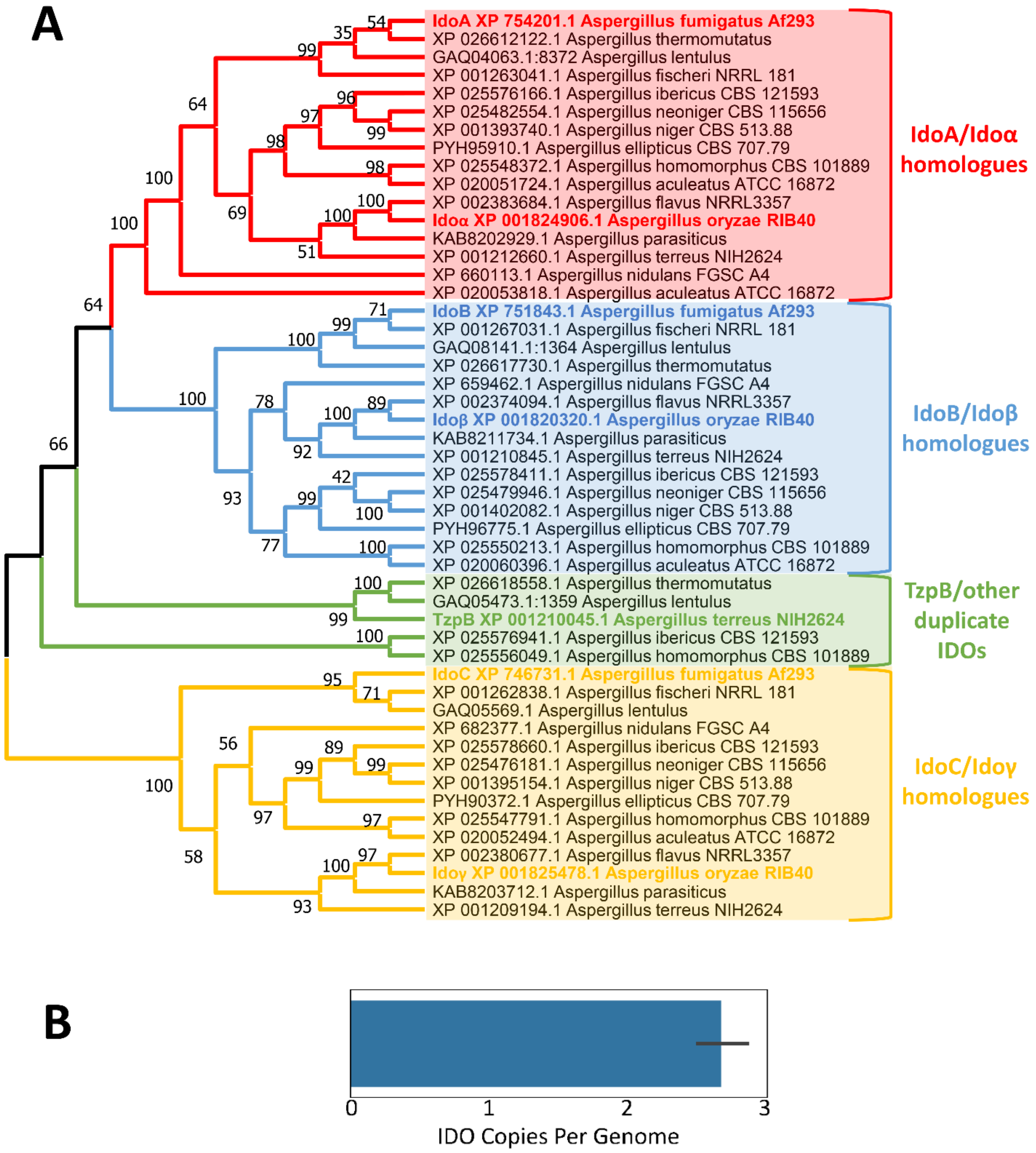

Figure S1. (a) Phylogenetic Tree of IDOs in a subset of Aspergilli. *IdoA*, *idoB*, and *idoC* homologs form distinct clades, as annotation according to reference sequences from *A. fumigatus* and *A. oryzae*. Interestingly, *tzpB* and other duplicated IDOs cluster together and share moderate sequence homology to both *idoA* and *idoB*. (b) average IDO counts in Aspergilli.
