# Supplementary material for: Heterologous Expression of the Unusual Terreazepine Biosynthetic Gene Cluster Reveals a Promising Approach for Identifying New Chemical Scaffolds
Source: mBio. 2020 Aug 25;11(4):e01691-20. doi: 10.1128/mBio.01691-20 (PMC7448278; doi:10.1128/mBio.01691-20)
Supplement: TABLE S1 [file mBio.01691-20-st001.pdf]

Table S1A. Annotated Boundaries of AtFAC7O19 in comparison with the *A. terreus* NIH2624 reference genome.

AtFAC7O19				NIH strain 2624-reference genome			
Gene ID	Start	End	Annotation	Gene ID	Start	End	Annotation
FAC38_01	3638	0	hypothetical protein	ATEG_07322	1348372	1344737	conserved hypothetical protein
FAC38_02	6960	5719	ER membrane protein complex subunit 1	ATEG_07323	1352787	1350457	conserved hypothetical protein
FAC38_03	8050	7141	hypothetical protein				
FAC38_04	9319	10876	Fumitremorgin C synthase	ATEG_07324	1354050	1355480	predicted protein
FAC38_06	15202	13926	Putative metal ion transporter C17A12.14	ATEG_07325	1359921	1358646	conserved hypothetical protein
FAC38_07	15808	17277	Pantothenate transporter liz1	ATEG_07326	1360528	1361996	conserved hypothetical protein
FAC38_08	19341	17338	Fatty acid amide hydrolase	ATEG_07327	1364050	1362058	conserved hypothetical protein
FAC38_09	20883	19695	3-methylitaconate isomerase	ATEG_07328	1365585	1364398	conserved hypothetical protein
FAC38_10	21283	22219	hypothetical protein	ATEG_07329	1365986	1366921	conserved hypothetical protein
FAC38_11	24418	25889	Gamma-glutamylputrescine oxidoreductase	ATEG_07330	1367765	1369235	predicted protein
FAC38_12	26261	27728	IMP-specific 5'-nucleotidase 1	ATEG_07331	1369611	1371078	IMP-specific 5'-nucleotidase 1
FAC38_13	30440	27974	ATP-dependent 6-phosphofructokinase 1	ATEG_07332	1373797	1371331	6-phosphofructokinase alpha subunit
FAC38_14	31051	33061	Mediator of RNA polymerase II transcription subunit 17	ATEG_07333	1374409	1376418	conserved hypothetical protein
FAC38_15	35119	33296	Quinate permease	ATEG_07334	1378469	1376648	conserved hypothetical protein
FAC38_16	37800	36201	putative transporter C1039.04	ATEG_07335	1381160	1379562	hypothetical protein
FAC38_17	41073	39841	Alcohol dehydrogenase 4, mitochondrial	ATEG_07336	1384425	1383205	conserved hypothetical protein
FAC38_18	43019	41573	Mannan endo-1,6-alpha-mannosidase DCW1	ATEG_07337	1386373	1384927	conserved hypothetical protein
FAC38_19	44957	46671	Acetamidase	ATEG_07338	1388362	1390075	similar to general amidase-C
FAC38_20	51279	52326	Alkylglycerol monooxygenase	ATEG_07340	1394631	1395677	conserved hypothetical protein
FAC38_21	54059	55756	Bifunctional solanapyrone synthase	ATEG_07341	1397419	1399120	predicted protein
FAC38_22	58509	55975	Lysine/arginine permease	ATEG_07342	1401880	1399340	conserved hypothetical protein
FAC38_23	59726	61214	Protein unc-13 homolog C	ATEG_07343	1403091	1404096	predicted protein
FAC38_24	64500	61508	G-type lectin S-receptor-like serine/threonine-protein kinase RLK1	ATEG_07344	1407863	1404869	predicted protein
FAC38_25	68806	67005	Dimethyl-sulfide monooxygenase	ATEG_07345	1412174	1410203	similar to monoxygenase
FAC38_26	69886	72284	putative urea active transporter 1	ATEG_07346	1413270	1415658	conserved hypothetical protein
FAC38_27	73426	72667	Uncharacterized protein AFUA_5G13800	ATEG_07347	1416653	1416039	conserved hypothetical protein
FAC38_28	74482	75130	hypothetical protein	none			
FAC38_29	75731	75995	Follistatin-related protein 4	none			
FAC38_30	78292	79862	Ankyrin-3	none			
FAC38_31	83202	81611	Zinc-type alcohol dehydrogenase-like protein C1198.01	ATEG_07354	1438182	1436592	zinc-binding dehydrogenase family oxidoreductase, putative
FAC38_32	87248	85539	High-affinity glucose transporter HXT2	ATEG_07355	1442234	1440526	sugar transporter

FAC38_33	87745	89287	Beta-fructofuranosidase, insoluble isoenzyme CWINV1	ATEG_07356	1442733	1444277	glycoside hydrolase family 32 protein
FAC38_34	91171	89323	Transcriptional activator protein DAL81	ATEG_07357	1446159	1445022	fungal specific transcription factor domain-containing protein
FAC38_35 **(TzpA)**	99708	92114	Nonribosomal peptide synthetase	ATEG_07358	1454709	1447102	ACAC, nonribosomal peptide synthase
FAC38_36 **(TzpB)**	102627	101177	Indoleamine 2,3-dioxygenase	ATEG_07359	1457658	1456205	indoleamine 2,3-dioxygenase family protein*
FAC38_37	105715	106234	Thaumatin-like protein	ATEG_07360	1460750	1461268	extracellular thaumatin domain protein
FAC38_38	108331	107028	Anhydro-N-acetylmuramic acid kinase	ATEG_07361	1463361	1462050	integral membrane protein
FAC38_39	109165	110878	Fumitremorgin C synthase	ATEG_07362	1464263	1465894	cytochrome P450
FAC38_40	111629	113101	hypothetical protein	ATEG_07363	1466644	1468115	MFS transporter
FAC38_41	113890	114880	Kinesin light chain	ATEG_07364	1468907	1469896	hypothetical protein

Table S1B. Primers and oligonucleotides used to produce gene and NRPS-domain deletions.

Name	Sequence (5'→3')
At7O19ΔC/T-gk-Fwd	ATAATCGAAAGTATCATTGCTTGCATCTCAGCCACTAGCTCGTCTAT**CTA**cctgttgacaattaatcatcggca
At7O19ΔT-Ap-Rev	AAGAGCAAAAGCGAGTCTGGGAGCCGTCCTTGTCTGCCGCTGTCGTTCGTtcagccaatcgactggcgagcgg
At7O19ΔT-Ap-Rev	TGACCAAAACCAACAATCGCTTGGAAAGTTTTCGCGGAACGCTGGCGCGCtcagccaatcgactggcgagcgg
At7O19-58ΔT-oligo	CGAAAGTATCATTGCTTGCATCTCAGCCACTAGCTCGTCTAT**CTA**ACGAACGACAGCGGCAGACAAGGACGGC TCCCAGACTCGCTTTTG
At7O19-58ΔCT-oligo	CGAAAGTATCATTGCTTGCATCTCAGCCACTAGCTCGTCTAT**CTA**GCGCGCCAGCGTTCCGCGAAAACTTTCCA AGCGATTGTTGGTTTT
At7O19-OE::TF-gpd-Fwd	TCCTCATGAATTAGATGGTTAGATGGACCTACCATCAGGATAGGTTCCATtgtgatgtctgctcaagcggg
At7O19-OE::TF-gk-Rev	TGGGACTTTGTCGCTCACGATTCGCCGAGTTGTATGGGCTGACCAGTGACtcagcactgtcctgctcctt
At7O19ΔtzpA-k-Fwd	CTCATAATCGGAAGTATCATTGCTTGCATCTCAGCCACTAGCTCGTCTATcgacctgcagcctgttga
At7O19ΔtzpA-k-Rev	GATTCATCAGAATACCTCTTTCGCCCAGCTCTCATTGTCGCGAAATCAATgtcgaggctgatcagcga
At7O19ΔtzpB-k-Fwd	TTTTGAATTTTATGGGGCACAGAAACATTTCAAATAAAACAGAATGAGATcgacctgcagcctgttga
At7O19ΔtzpB –k-Rev	ATGGACCTTTGCCTCGTGGATTGACAGGTCCACCGTGGATGAGAACGATCgtcgaggctgatcagcga

Bold: stop codon, lower case: primers for selection markers, gk: GalK gene, Ap: Apramycin resistance gene, k: Kanamycin gene, gpd: gpdA promoter
