# Supplementary material for: Heterologous Expression of the Unusual Terreazepine Biosynthetic Gene Cluster Reveals a Promising Approach for Identifying New Chemical Scaffolds
Source: mBio. 2020 Aug 25;11(4):e01691-20. doi: 10.1128/mBio.01691-20 (PMC7448278; doi:10.1128/mBio.01691-20)
Supplement: FIG S2 [file mBio.01691-20-sf002.pdf]

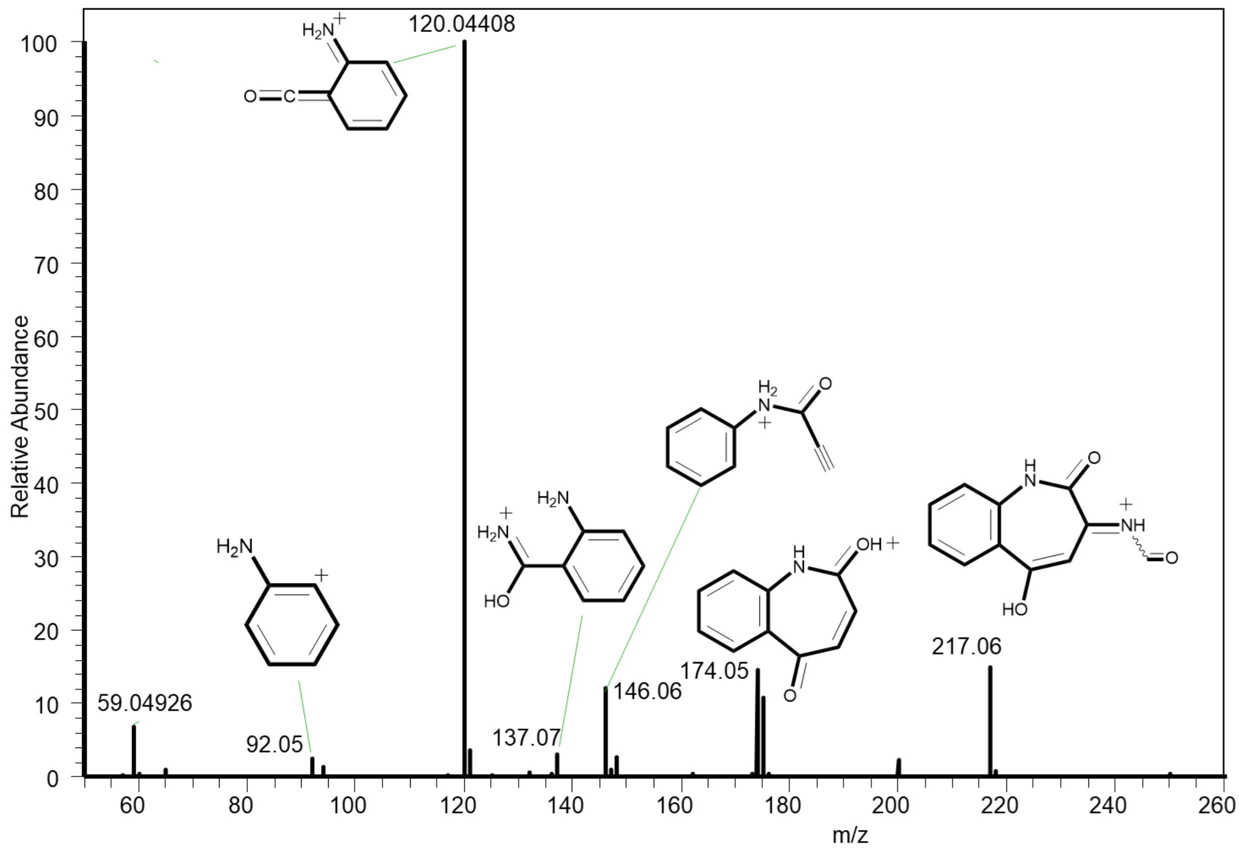

Figure S2. MS^2 fragmentation spectra for terreazepine, fragmented through HCD at a normalized collision energy of 25.0%.
