# Supplementary material for: Heterologous Expression of the Unusual Terreazepine Biosynthetic Gene Cluster Reveals a Promising Approach for Identifying New Chemical Scaffolds
Source: mBio. 2020 Aug 25;11(4):e01691-20. doi: 10.1128/mBio.01691-20 (PMC7448278; doi:10.1128/mBio.01691-20)
Supplement: FIG S3 [file mBio.01691-20-sf003.pdf]

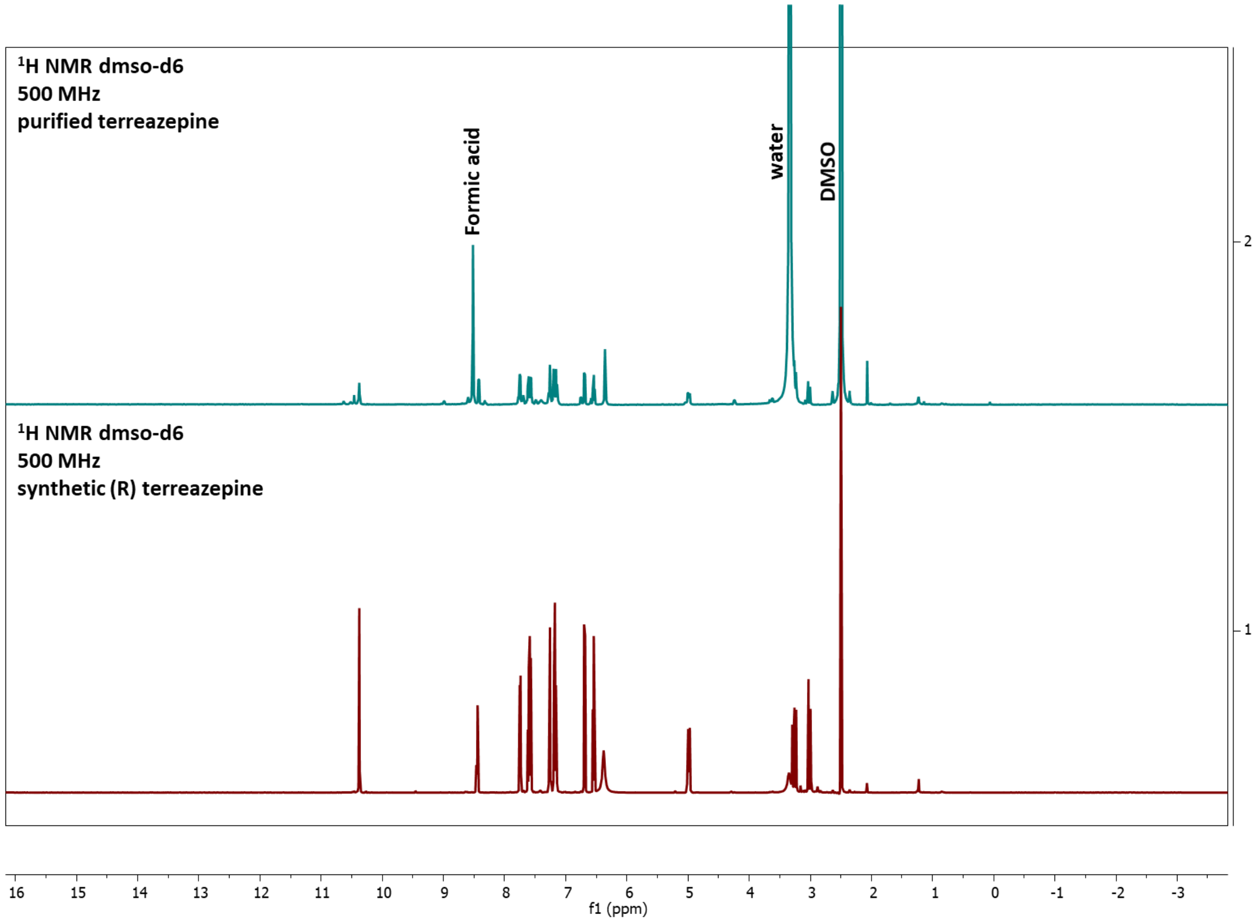

Figure S3A. Overlapping ^{1}H NMR spectra for natural (top) and synthetic (bottom) terreazepine in DMSO-d_6. ^{1}H signals are consistent between samples, indicating that the correct product was obtained through synthesis.

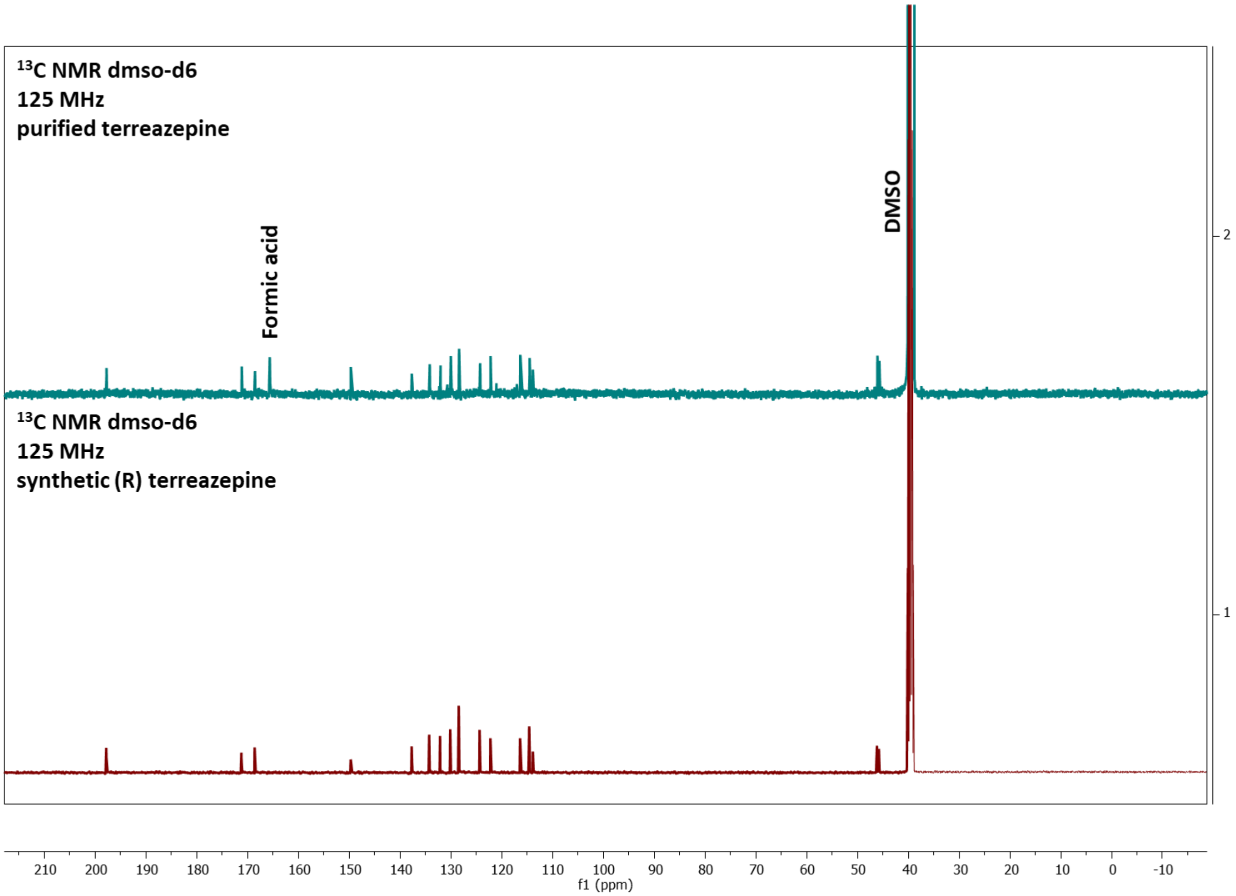

Figure S3B. Overlapping ^{13}C NMR spectra for natural (top) and synthetic (bottom) terreazepine in DMSO-d_6. ^{13}C signals are consistent between samples, indicating that the correct product was obtained through synthesis.

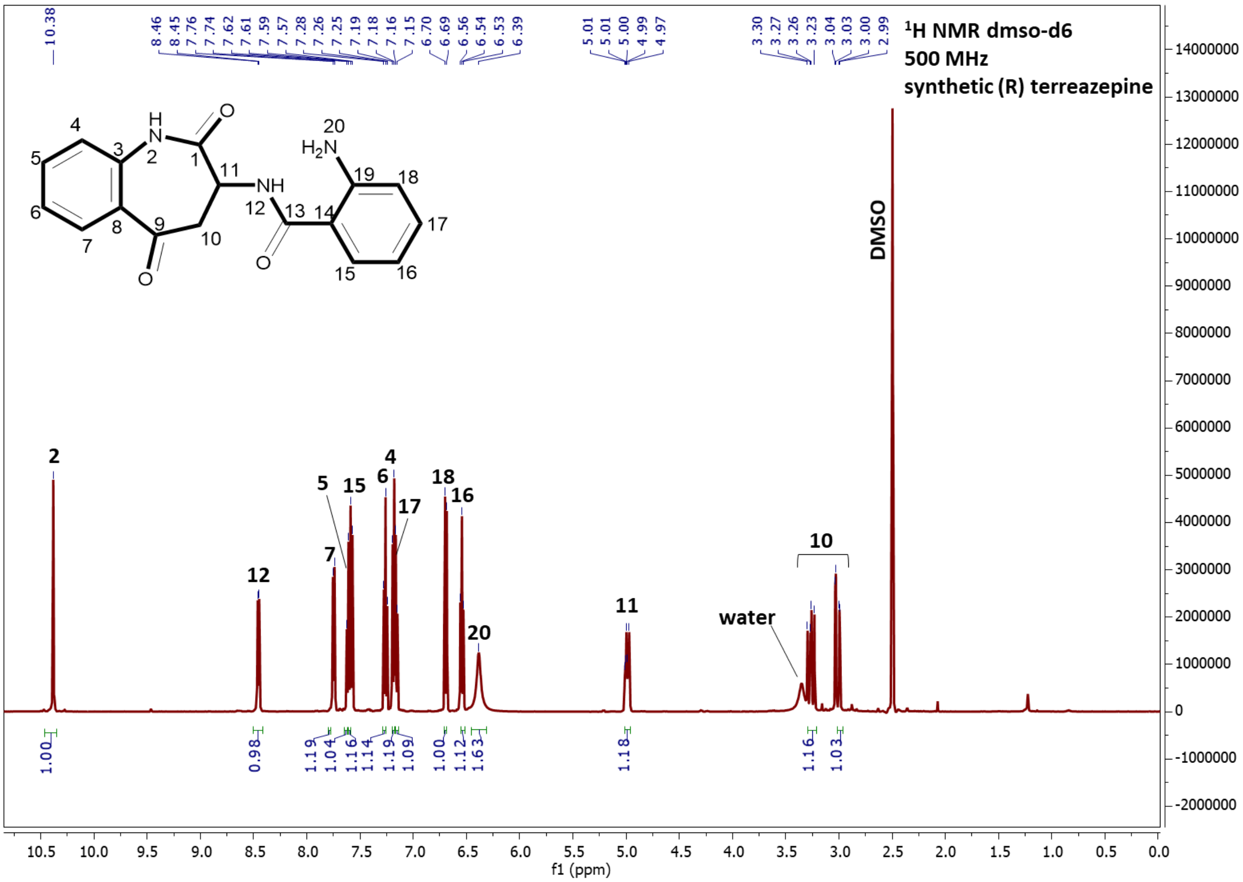

Figure S3C. ^{1}H NMR data for synthetic (R) terreazepine (500 MHz, DMSO-d_6).

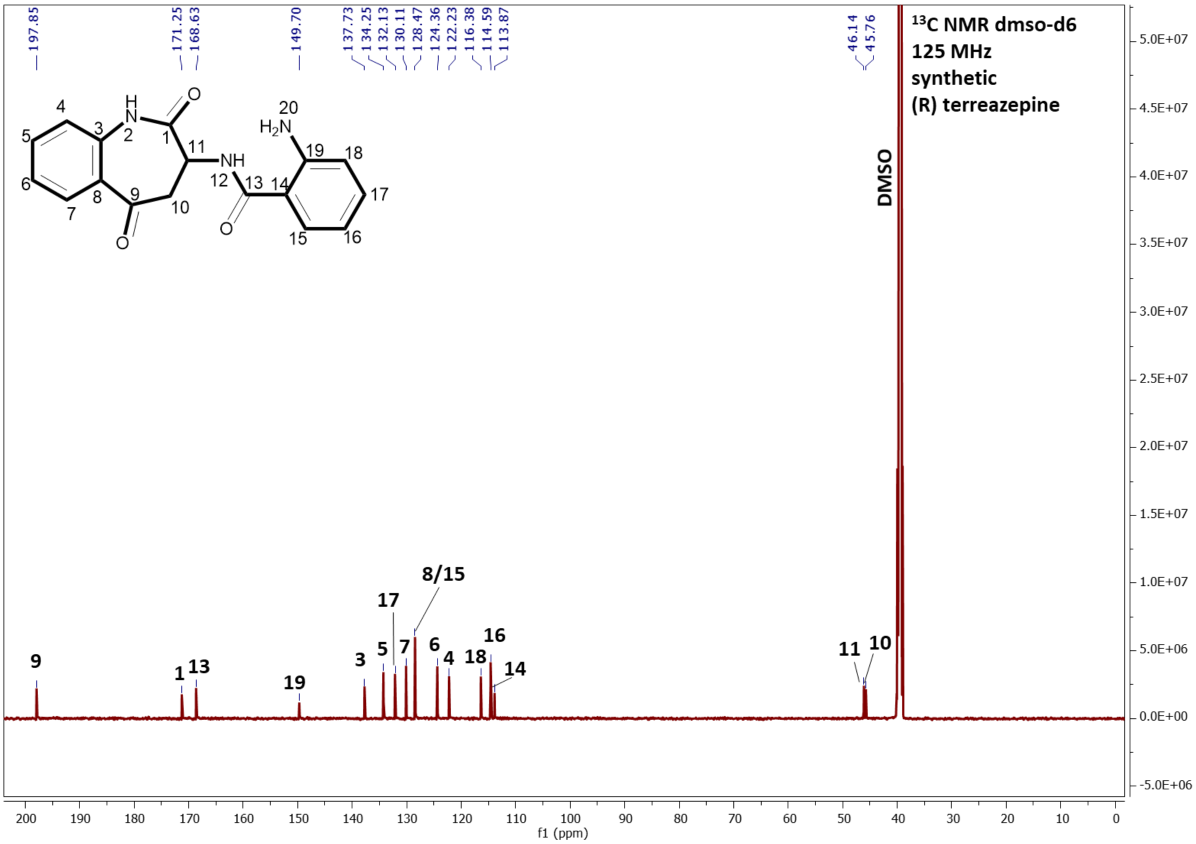

Figure S3D. ^{13}C NMR data for synthetic (R) terreazepine (125 MHz, DMSO-d_6).

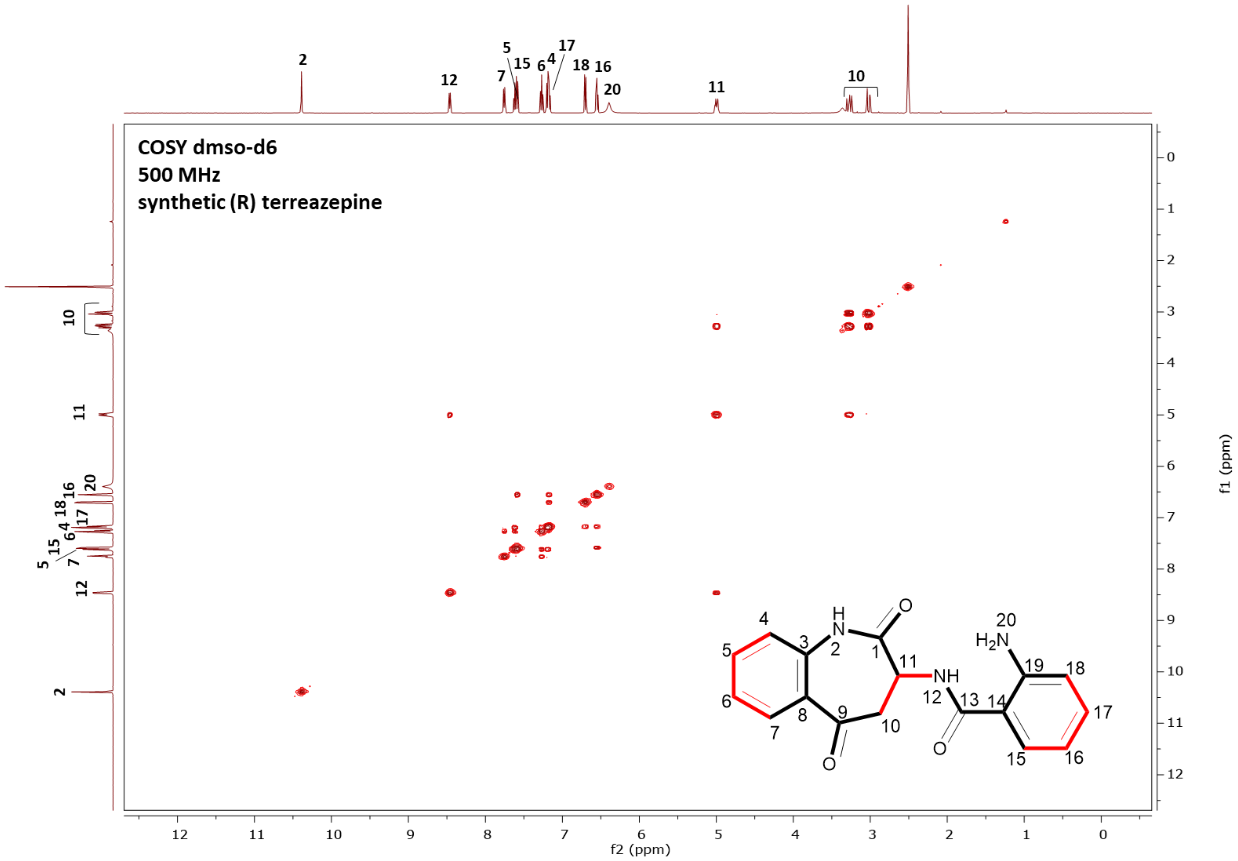

Figure S3E. COSY data for synthetic (R) terreazepine (500 MHz, DMSO-d_6).

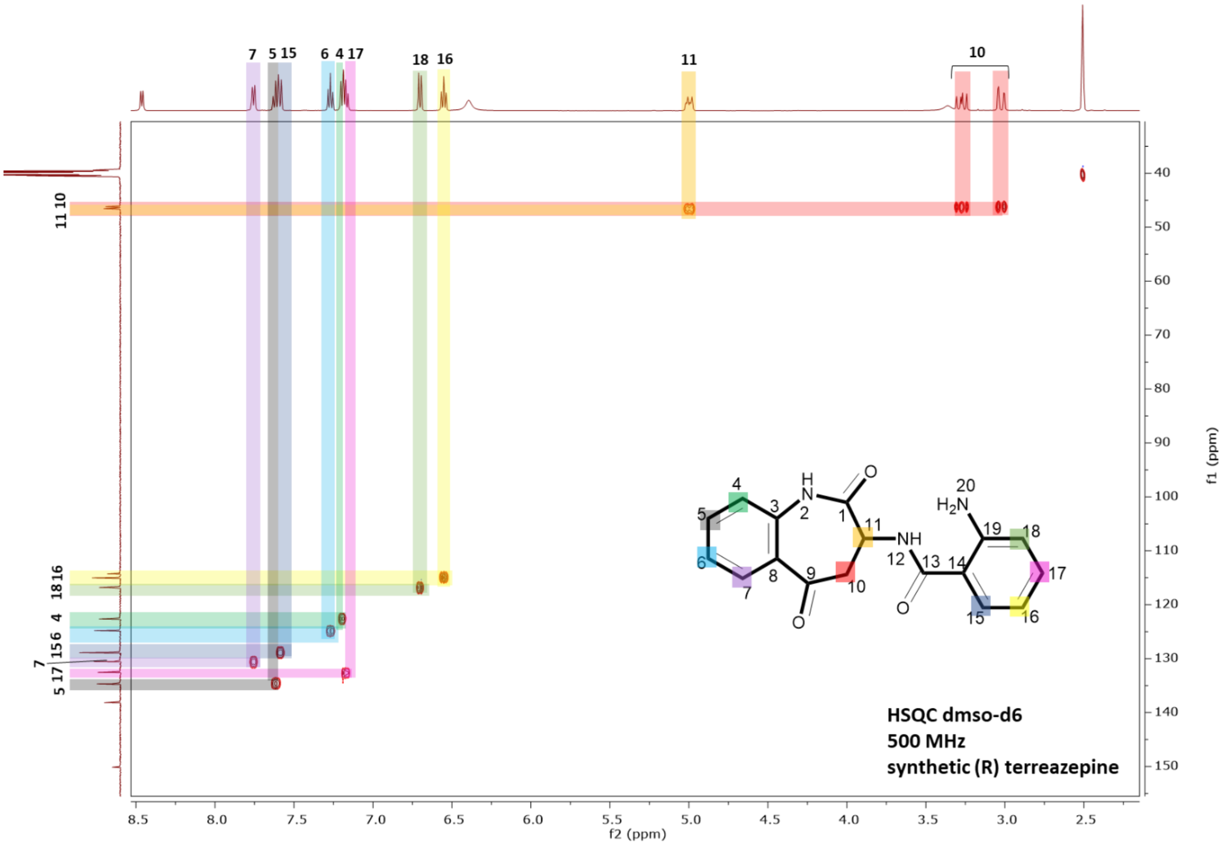

Figure S3F. HSQC data for synthetic (R) terreazepine (500 MHz, DMSO-*d₆*)

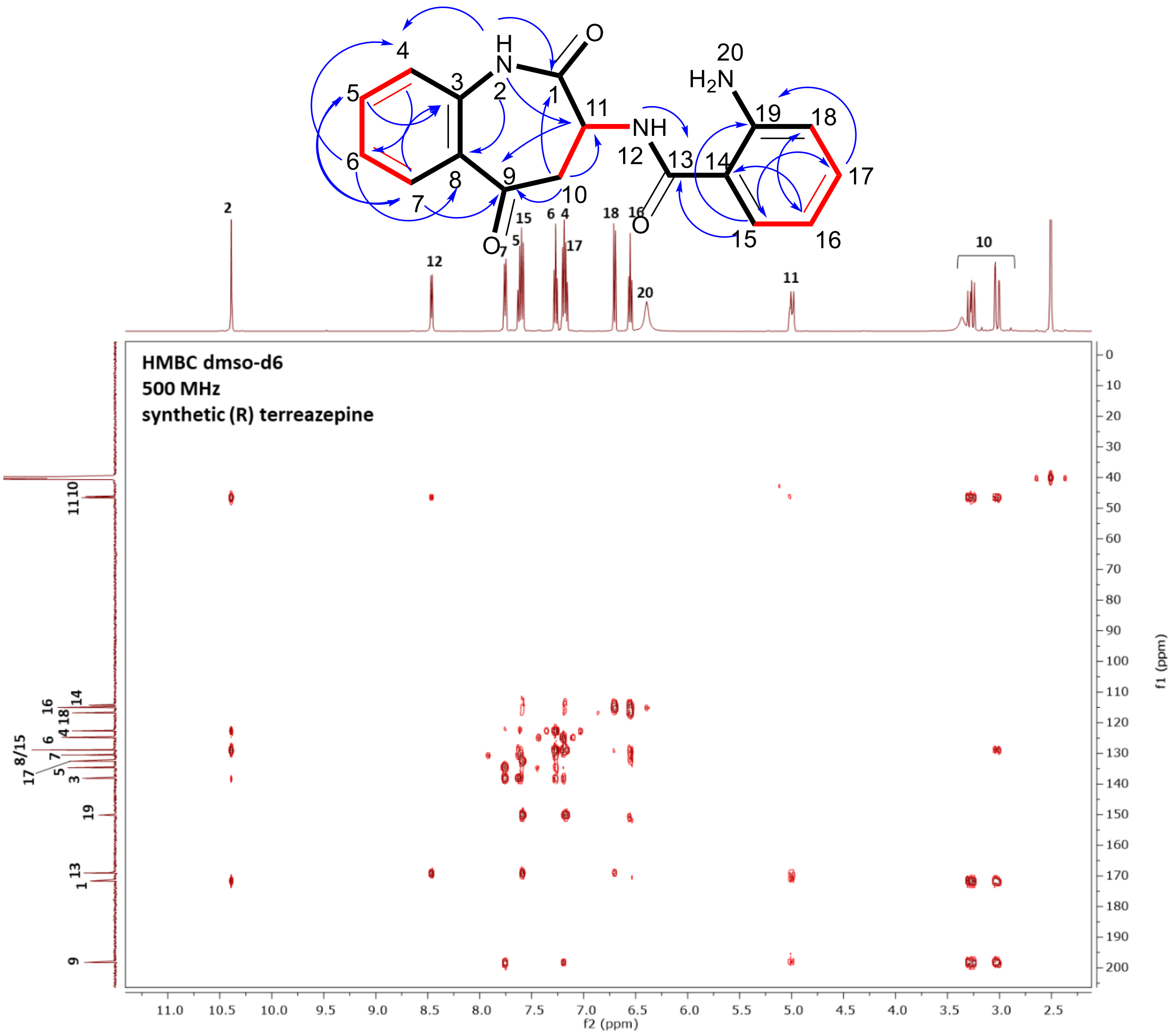

Figure S3G. HMBC data for synthetic (R) terreazepine (500 MHz, DMSO-d_6)

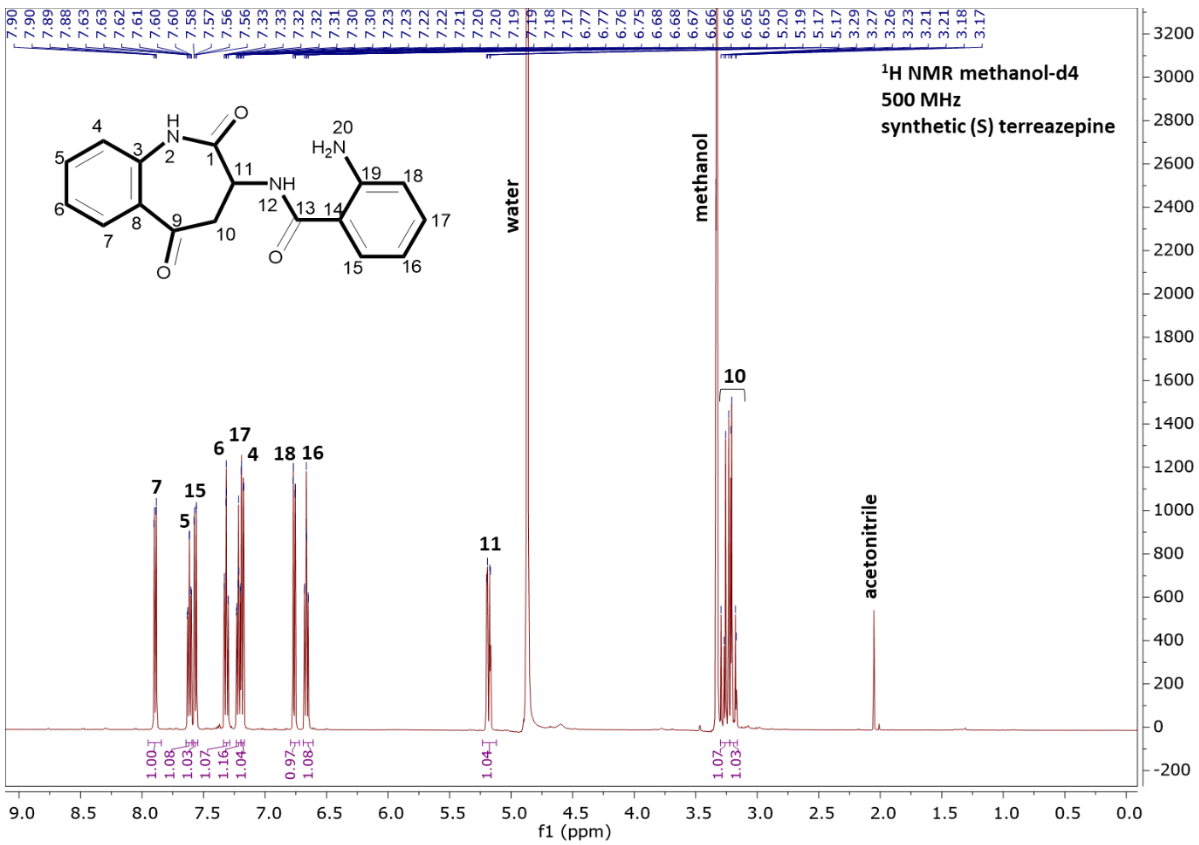

Figure S3H. ^{1}H NMR spectra for (S)-terreazepine in methanol-d_4 (500 MHz)

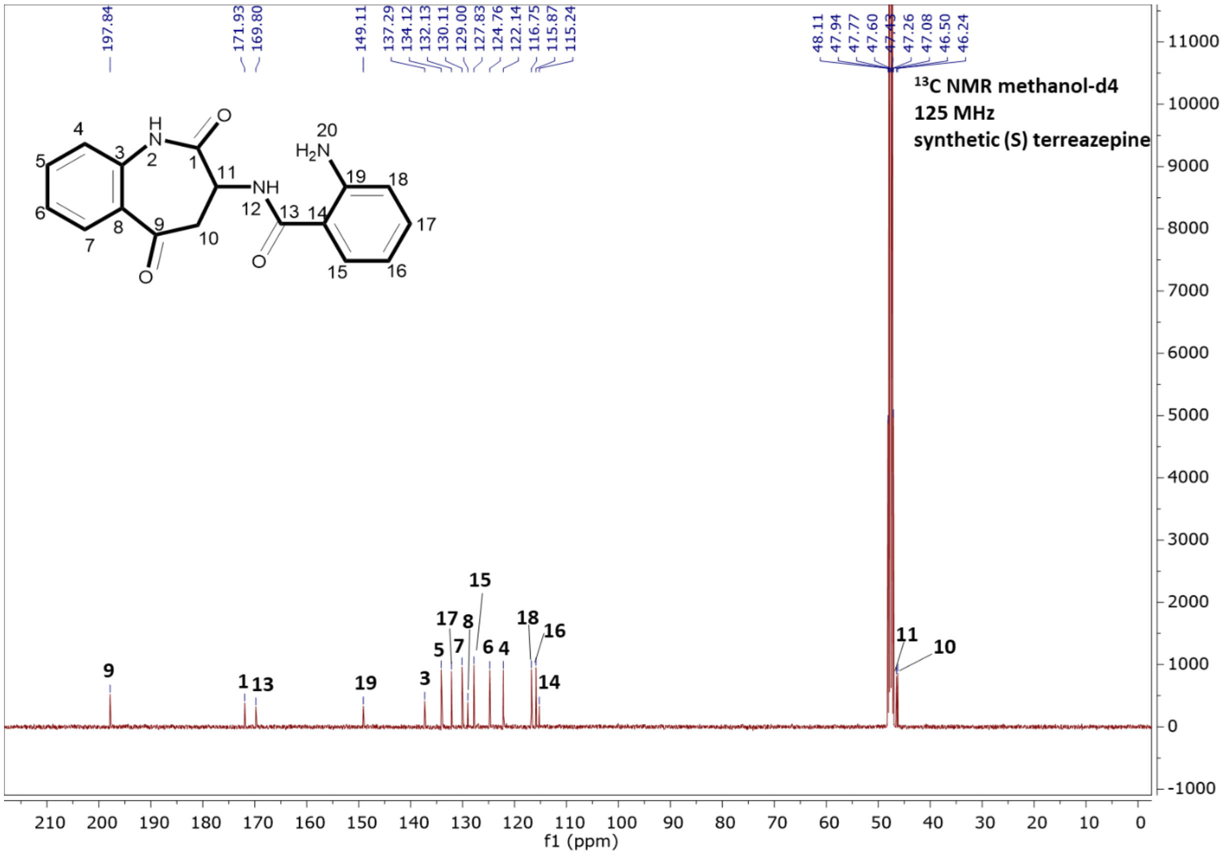

Figure S3I. ^{13}C NMR spectra for (S)-terreazepine in methanol-d_4 (125 MHz)
