# Supplementary material for: Heterologous Expression of the Unusual Terreazepine Biosynthetic Gene Cluster Reveals a Promising Approach for Identifying New Chemical Scaffolds
Source: mBio. 2020 Aug 25;11(4):e01691-20. doi: 10.1128/mBio.01691-20 (PMC7448278; doi:10.1128/mBio.01691-20)
Supplement: FIG S4 [file mBio.01691-20-sf004.pdf]

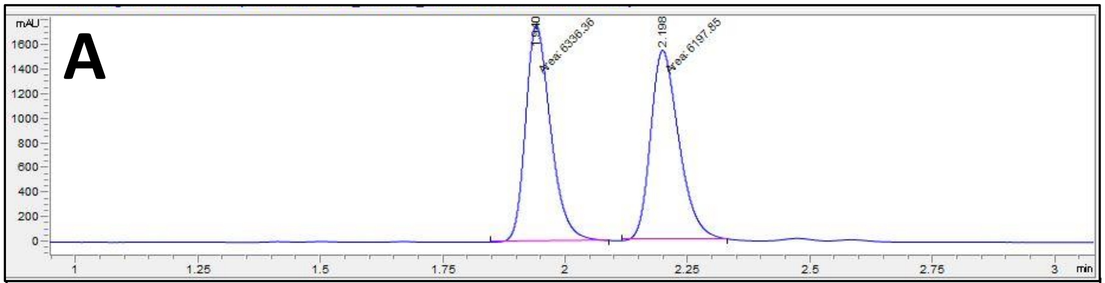

Time (min)	Type	Area	Height	Width	Area %	Symmetry
1.940	MM	6336.4	1762.7	0.0599	50.533	0.740
2.198	MM	6197.9	1546.4	0.0668	49.447	0.733

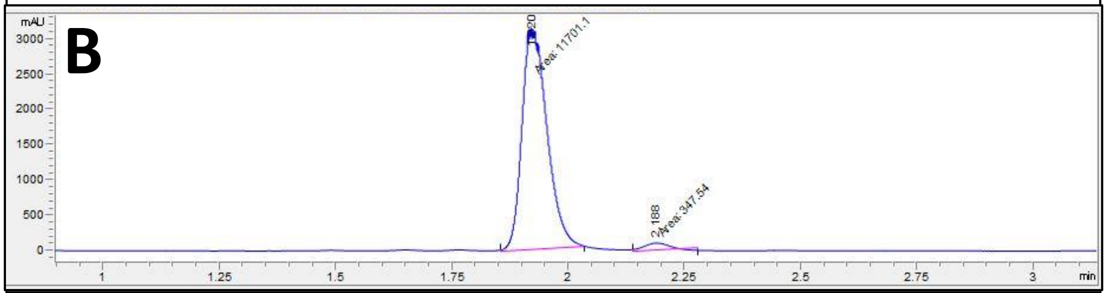

Time (min)	Type	Area	Height	Width	Area %	Symmetry
1.920	MM	11701.1	3130.2	0.0623	97.116	0.596
2.188	MM	347.5	98.3	0.0589	2.884	1.25

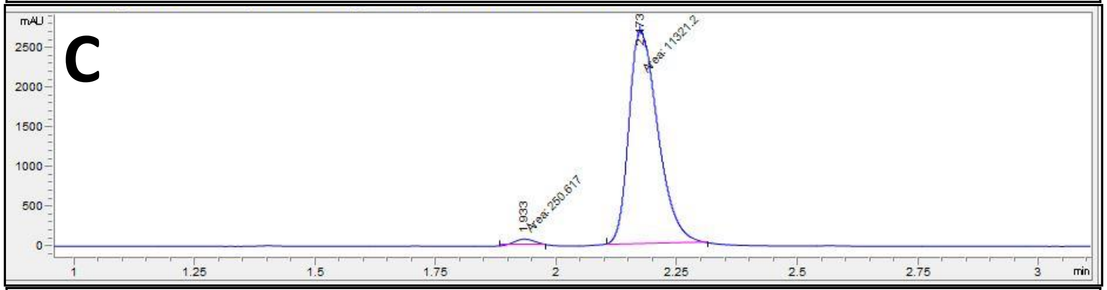

Time (min)	Type	Area	Height	Width	Area %	Symmetry
1.933	MM	250.6	81.5	0.0513	2.166	0.905
2.173	MM	11321.2	2762.5	0.0683	97.834	0.59

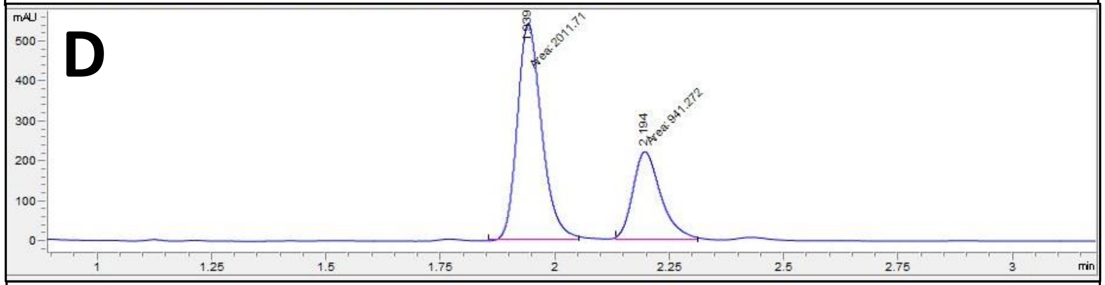

Time (min)	Type	Area	Height	Width	Area %	Symmetry
1.939	MM	2011.7	544.3	0.0616	68.125	0.791
2.194	MM	941.3	221.3	0.0709	31.875	0.740

Figure S4. SFC Results for (a) the acylated terreazpine racemic mixture, (b) acylated synthetic (S)-enantiomer, (c) acylated synthetic (R)-enantiomer, (d) and acylated natural terreazepine.
