# Supplementary material for: Heterologous Expression of the Unusual Terreazepine Biosynthetic Gene Cluster Reveals a Promising Approach for Identifying New Chemical Scaffolds
Source: mBio. 2020 Aug 25;11(4):e01691-20. doi: 10.1128/mBio.01691-20 (PMC7448278; doi:10.1128/mBio.01691-20)
Supplement: TABLE S2 [file mBio.01691-20-st002.pdf]

Table S2. NMR data for terreazepine in DMSO-*d6.* ^{1}H, COSY, HMBC, and HSQC data collected at 500 MHz, and ^{13}C data collected at 125 MHz. Overlapping assignments (*) were determined using HSQC and HMBC data.

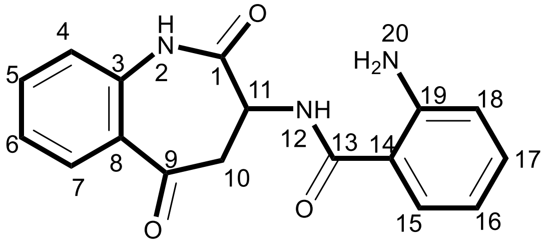

Position	^{13}C	^{1}H	HMBC	COSY
**1**	171.25	-	2, 10α, 10β	-
**2**	-	10.38, s, 1H	-	-
**3**	137.72	-	5,7	-
**4**	122.23	7.19, d, J=8.0, 1H	2, 6	5
**5**	134.25	7.61, td, J=7.24, 1.42, 1H	7	4,6
**6**	124.36	7.27, t, J=7.57, 1H	4	7,5
**7**	130.12	7.76, dd, J=7.88, 1.67, 1H	5	6
**8**	128.47*	-	2,6,10α	-
**9**	197.76	-	7, 10α, 10β, 11	-
**10**	45.75	10α: 3.02, dd, J=18.7, 2.6, 1H<br>10β: 3.24, dd, J=18.7, 13.3, 1H	-	11
**11**	46.14	4.99, ddd, J=13.2, 7.4, 2.5, 1H	2, 10α, 10β	10β,12
**12**	-	8.42, d, J=7.42, 1H	-	11
**13**	168.63	-	12,15	-
**14**	113.87	-	16	-
**15**	128.47*	7.58, d, J=7.6, 1H	17	16
**16**	114.59	6.54, t, J=7.9, 1H	18	15,17
**17**	132.13	7.17, m, 1H	15	16, 18
**18**	116.36	6.69, d, J=8.1, 1H	16	-
**19**	149.70	-	15,17	-
**20**	-	6.38, s, 2H	-	-
