# Supplementary material for: Heterologous Expression of the Unusual Terreazepine Biosynthetic Gene Cluster Reveals a Promising Approach for Identifying New Chemical Scaffolds
Source: mBio. 2020 Aug 25;11(4):e01691-20. doi: 10.1128/mBio.01691-20 (PMC7448278; doi:10.1128/mBio.01691-20)
Supplement: FIG S5 [file mBio.01691-20-sf005.pdf]

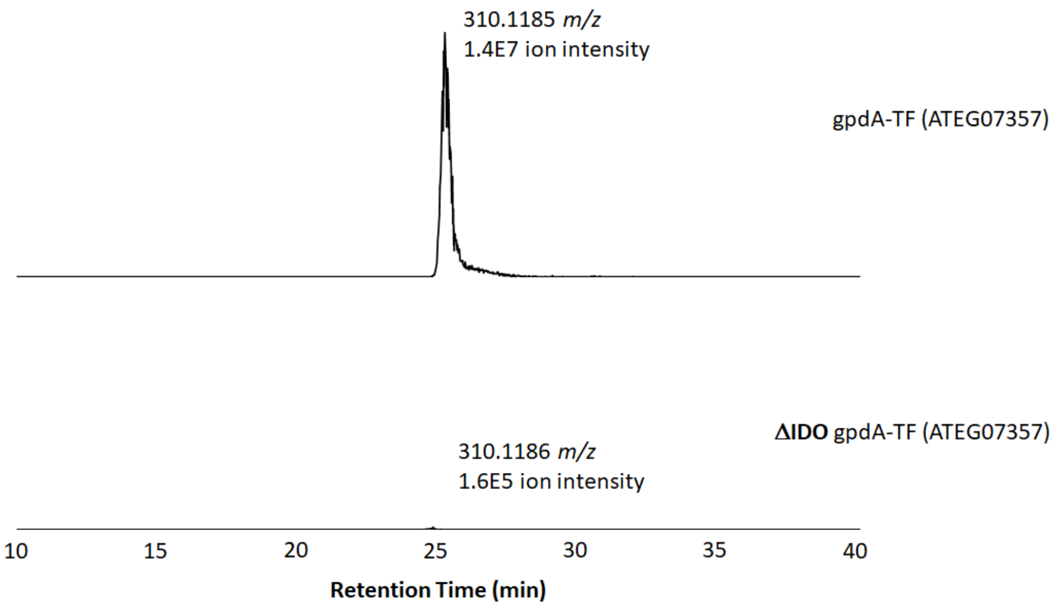

Figure S5. Selected ion chromatograms of terreazepine in FAC control (top) and *tzpB* deletion mutants (bottom). The very low production of terreazepine in the deletant strain confirms the involvement of the IDO in terreazepine production.
