# Supplementary material for: Heterologous Expression of the Unusual Terreazepine Biosynthetic Gene Cluster Reveals a Promising Approach for Identifying New Chemical Scaffolds
Source: mBio. 2020 Aug 25;11(4):e01691-20. doi: 10.1128/mBio.01691-20 (PMC7448278; doi:10.1128/mBio.01691-20)
Supplement: TABLE S3 [file mBio.01691-20-st003.pdf]

Table S3. Adenylation domain substrate predictions for TzpA, a nonribosomal peptide synthetase and C_2, T_2, and T_3 domain active site sequence alignments. (**A**) TzpA-A_1 substrate binding residues bear similarity to many additional anthranilate-activating adenylation domains. Additionally, adenylation domains from *A. thermomutatus* (RHZ670305-A_1) and *A. lentulus* (GAQ05471-A_1) have an identical A domain sequence to that of TzpA-A_1, suggesting they also bind anthranilate. (**B**) TzpA-A_2 possesses a specificity sequence that is disparate from known kynurenine-binding A domains. It does, however, bear resemblance to the A_2 domains from the orphan NRPSs RHZ670305-A_2, and GAQ05471-A_2, and may represent a new type of kynurenine-activating adenylation domain. (**C**) The C_2 domain of TzpA does possess the catalytic histidine purported to be required for activity (J.A. Baccile, H.H. Le, B.T. Pfannenstiel, J.W. Bok, C. Gomez, E. Brandenburger, D. Hoffmeister, N.P. Keller, F.C. Schroeder, Angew Chem Int 58:14589-14593, 2019), although the remainder of its sequence diverges from other C_2 domains part of NRPSs with the ATCATCT domain architecture such as GliP and HasD. (**D**) The T_2 and T_3 domains of TzpA both appear functional when compared to GliP T domains and GrsA T domains with known functionality, (G.L. Challis, J. Ravel, C.A. Townsend, Chem Biol 7:211-224, 2000) given their sequence similarity and the presence of a conserved serine in the sequence. Residues are colored according to the Taylor coloring scheme (W.R. Taylor. Protein Engineering, Design, and Selection 10:743-746, 1997).

A

NRPS	Substrate	Specificity Code
**TzpA-A1**	Anthranilate (proposed)	G - I - I - L - F - G - V - V - T - K
Chrysogine synthetase (ADY16697)	Anthranilate	G - V - I - F - M - A - A - G - V - K
Benzomalvin synthetase (KX449366)	Anthranilate	G - I - N - F - I - G - A - G - T - K
Fumiquinazoline synthetase (EAL89049)	Anthranilate	G - V - I - I - L - A - A - G - I - K
Acetylaszonalenin synthetase (EAW16180)	Anthranilate	G - A - L - F - F - A - A - G - V - K
Chrysogine synthetase (ADY16697)	Anthranilate	G - V - I - F - M - A - A - G - V - K
RHZ67305-A1	Unknown	G - I - I - L - F - G - V - V - T - K
GAQ05471-A1	Unknown	G - I - I - L - F - G - V - V - T - K

B

NRPS	Substrate	Specificity Code
**TzpA-A2**	Kynurenine (proposed)	D - A - A - M - I - M - G - I - A - K
nidulanin synthetase (CBF87869)	Kynurenine	D - V - L - S - F - G - A - S - L - K
Daptomycin synthetase (AAX31559)	Kynurenine	D - A - W - T - T - T - G - V - G - K
Taromycin synthetase (AHH53508)	Kynurenine	D - A - W - T - T - T - G - V - A - K
RHZ67305-A1	Unknown	D - C - G - M - S - M - G - V - G - K
GAQ05471-A1	Unknown	D - C - G - M - S - M - G - V - G - K

C

C_2 Domain Active Site			
GliP-C_2 (EAL88817)	1753	S - H - A - V - A - D - L - N - S	1761
HasD-C_2 (EAL92291)	1789	S - H - V - V - G - D - A - A - T	1797
TzpA-C_2 (EAU32742)	2136	T - H - A - L - W - D - G - G - P	2144

D

T Domain Ppant Binding Site			
GrsA-T (BAA00406)	566	F - Y - A - L - G - G - D - S - I - K - A - I	577
GliP-T_2 (EAL88817)	1575	F - R - A - L - G - G - H - S - V - L - Q - M	1586
GliP-T_3 (EAL88817)	2088	F - F - E - A - G - G - D - S - I - Q - A - W	2099
TzpA-T_2 (EAU32742)	1930	F - F - H - L - G - G - D - S - V - N - G - M	1941
TzpA-T_3 (EAU32742)	2466	F - F - R - L - G - G - N - S - V - R - A - L	2477
