# Supplementary material for: Heterologous Expression of the Unusual Terreazepine Biosynthetic Gene Cluster Reveals a Promising Approach for Identifying New Chemical Scaffolds
Source: mBio. 2020 Aug 25;11(4):e01691-20. doi: 10.1128/mBio.01691-20 (PMC7448278; doi:10.1128/mBio.01691-20)
Supplement: FIG S6 [file mBio.01691-20-sf006.pdf]

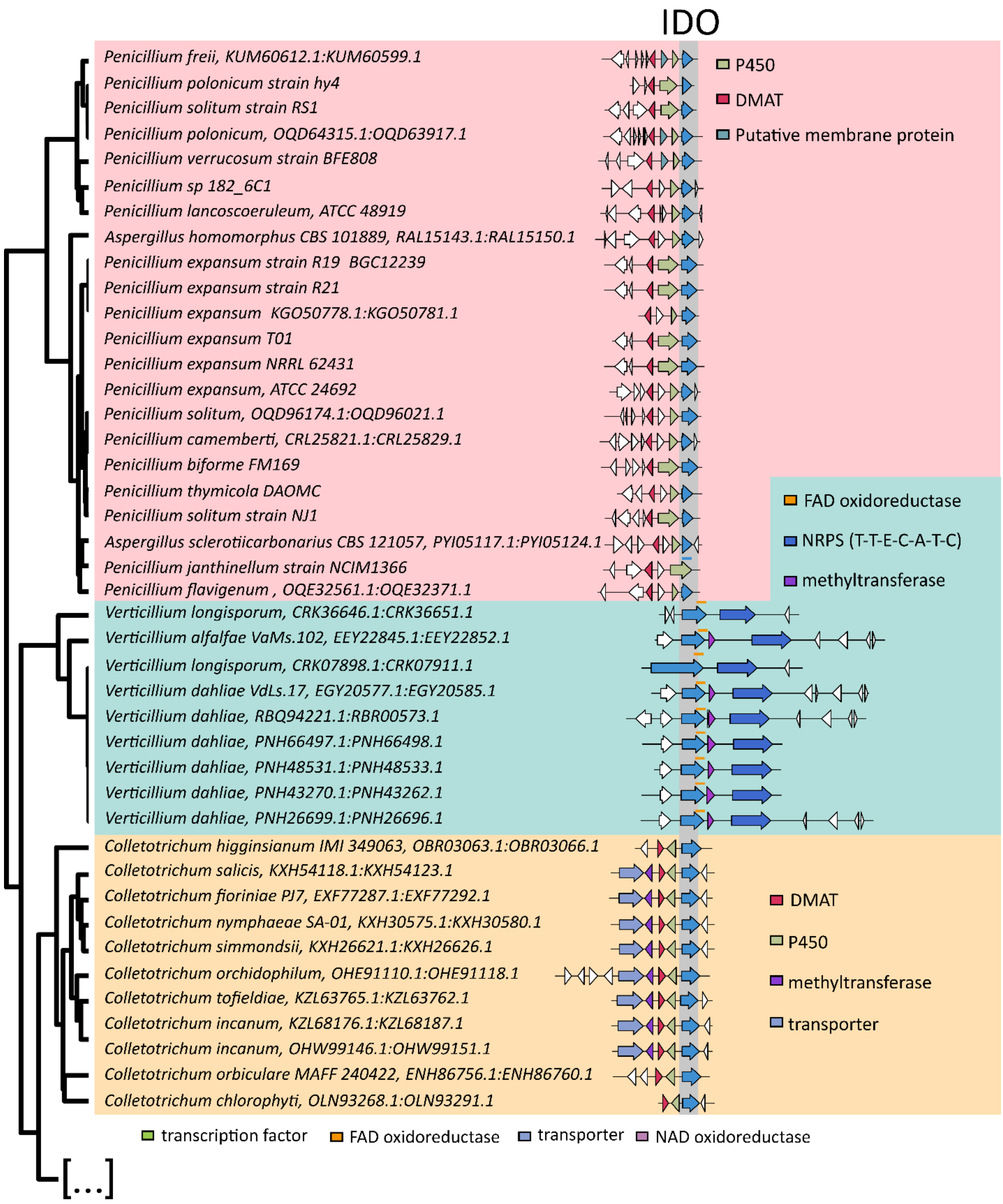


**Figure S6.** IDO-containing Biosynthetic Gene Clusters in Fungi. These gene clusters encompass a wide range of phylogenetically diverse fungi with diverse backbone gene domain sequences.

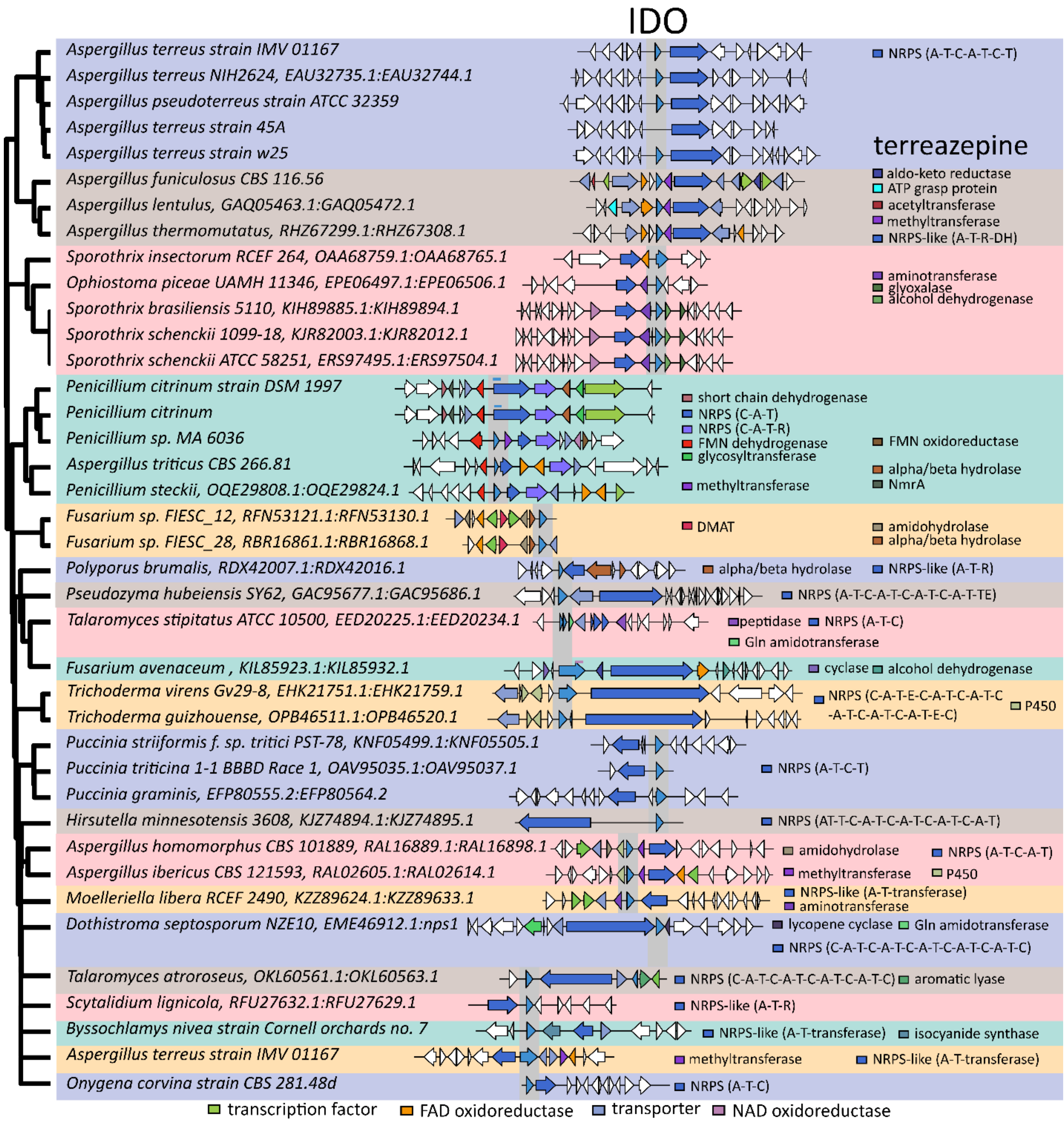


**Figure S6 (cont.).** IDO-containing Biosynthetic Gene Clusters in Fungi. These gene clusters encompass a wide range of phylogenetically diverse fungi with diverse backbone gene domain sequences.
