# Supplementary material for: Heterologous Expression of the Unusual Terreazepine Biosynthetic Gene Cluster Reveals a Promising Approach for Identifying New Chemical Scaffolds
Source: mBio. 2020 Aug 25;11(4):e01691-20. doi: 10.1128/mBio.01691-20 (PMC7448278; doi:10.1128/mBio.01691-20)
Supplement: TEXT S1 [file mBio.01691-20-s0001.docx]

Supplemental Materials and Methods for

**Heterologous expression of the unusual terreazepine biosynthetic gene cluster reveals a promising approach for identifying new chemical scaffolds**

Lindsay K. Caesar, Matthew T. Robey, Michael Swyers, Md N. Islam, Rosa Ye, Purav P. Vagadia, Gary E. Schiltz, Paul M. Thomas, Chengcang C. Wu, Neil L. Kelleher, Nancy P. Keller, Jin Woo Bok

Jin Woo Bok, Nancy P. Keller, Neil L. Kelleher, and Chengcang C. Wu

Emails: jbok@wisc.edu, npkeller@wisc.edu, n-kelleher@northwestern.edu, cwu@intactgenomics.com

**Experimental Methods:**

*Total Synthesis and Stereochemical Analysis of Terreazepine*

General Experimental Procedures.

All chemical reagents were obtained from commercial suppliers and used without further purification unless otherwise stated. Anhydrous solvents were purchased from Sigma-Aldrich. Reversed phase preparative HPLC was performed with the following conditions: Phenomenex Kinetex C18 50 x 30 mm; 5 µm. The gradient consisted of 10-90 % acetonitrile:water with 0.1 % formic acid over 7 min, then 1 min at 100 % acetonitrile at a flow rate of 50 mL/min. NMR spectra for intermediates and final synthetic compounds were obtained at 298.2 K using a Bruker Avance III 500 MHz system equipped with a DCH CryoProbe. Chemical shifts were reported in ppm (*δ*) and were referenced using residual non-deuterated solvent as an internal standard (CDCl_3_ at 7.24 ppm for ^1^H NMR and 77.0 for ^13^C NMR. CD_3_OD at 3.33 ppm for ^1^H NMR and 47.6 for ^13^C NMR. DMSO-d_6_ at 2.52 ppm for ^1^H NMR and 39.9 ppm for ^13^C NMR). Enantioselectivity measurements were made on an Agilent 1290 Infinity SFC using Chiralpak IB-3 chiral stationary phase. Conditions: 35% MeOH in CO_2_, flow rate – 2.5 mL/min, λ = 250 nm.

Synthesis of (S)-terreazepine, (**5**)

To a vial containing (S)-2-amino-4-(2-aminophenyl)-4-oxobutanoic acid (100 mg, 1 equiv, 0.48 mmol, **1**) (L-Kynurenine) was added 1,4-Dioxane (2 mL) followed by triethylamine (63 mg, 87 µL, 1.3 equiv, 0.62 mmol). The mixture was sonicated briefly after which Boc_2_O (115 mg, 0.12 mL, 1.1 equiv, 0.53 mmol) was added and the suspension was stirred overnight at room temperature for 16 h after which the reaction mixture was clear. The reaction mixture was concentrated to provide compound **2** as a foam (148 mg, quant.) and it was taken on to the next step without further purification.

To compound **2** (148 mg, 1 equiv, 0.48 mmol) was added DMF (2 mL) and DIPEA (124 mg, 0.17 mL, 2 equiv, 0.96 mmol) after which HATU (237 mg, 1.3 equiv, 0.63 mmol) was added. The reaction mixture was stirred at room temperature for 30 min after which it was directly purified by RP HPLC eluting with 10 to 90 % acetonitrile in water (0.1 % formic acid). Relevant fractions were concentrated to give compound **3** (70 mg, 0.24 mmol, 50 %) as a brown solid. ^1^H NMR (500 MHz, Chloroform-*d*) δ 7.86 – 7.80 (m, 1H), 7.69 (s, 1H), 7.51 (td, *J* = 7.7, 1.6 Hz, 1H), 7.29 – 7.25 (m, 1H), 6.95 (d, *J* = 8.0 Hz, 1H), 5.66 (d, *J* = 6.5 Hz, 1H), 4.85 (ddd, *J* = 12.5, 6.3, 2.8 Hz, 1H), 3.30 (dd, *J* = 19.0, 3.0 Hz, 1H), 2.99 (dd, *J* = 19.0, 13.0 Hz, 1H), 1.42 (s, 9H).

Compound **3** (70 mg, 1 equiv, 0.24 mmol) was dissolved in DCM (1 mL) and hydrogen chloride (4 M in dioxanes) (1200 µL, 4 molar, 10 equiv, 4.80 mmol) was added after which the reaction mixture was stirred at room temperature for 4 h. The mixture was concentrated to give compound **4** (55 mg, quant., HCl salt).

Compound **4** (50 mg, 1 equiv, 0.22 mmol) was dissolved in DMF (1 mL) after which 2-aminobenzoic acid (30 mg, 1 equiv, 0.22 mmol) and DIPEA (71 mg, 96 µL, 2.5 equiv, 0.55 mmol) were added. Lastly, HATU (110 mg, 1.3 equiv, 0.29 mmol) was added and the reaction mixture was stirred at room temperature for 1 h after which the reaction mixture was directly purified by RP HPLC eluting with 10 to 90 % acetonitrile in water (0.1 % formic acid) to give (S)-terreazepine, compound **5** (40 mg, 0.13 mmol, 59 %, 97:3 er). Presumably due to the presence of the primary aromatic amine in compound **5**, SFC measurements on compound **5** produced a broad peak which was difficult to resolve. Therefore, the aniline was acylated as shown below and SFC measurements were completed using compound **6**. A racemic mixture/standard was made by creating a 1:1 mixture of **6** and its enantiomer **8**, which was synthesized separately (see next section).

Compound **5** (1 mg, 3 umol, 1.3 equiv) was dissolved in MeOH (1 mL) and acylated by adding acetic anhydride (30 uL, 0.3 mmol, 100 equiv) and stirring the mixture overnight at room temperature for 16 h after which acylation was complete by LCMS and no other peaks were observed. SFC measurements were taken on compound **6** using a Chiralpak IB-3 stationary phase, (35% MeOH in CO_2_, flow rate = 2.5 mL/min, λ = 250 nm): 1.92 min (major), 2.19 min (minor) (**Figure S4B**). ^1^H and ^13^C NMR spectra were confirmed in methanol-d4, and match shifts reported in the “*Purification and Structural Analysis of Terreazepine”* section (**Figures S3H-S3I**).

Synthesis of (R)-terreazepine (**7**)

In order to synthesize (R)-terreazepine, the same synthetic route and purification procedure as that of compound **5** were utilized. Starting with 100 mg of (R)-2-amino-4-(2-aminophenyl)-4-oxobutanoic acid (D-Kynurenine), the four step synthetic route and purification protocol yielded (R)-terreazepine, compound **7** (40 mg, 98:2 er). SFC separation: Chiralpak IB-3 stationary phase, (35 % MeOH in CO2, flow rate = 2.5 mL/min, λ = 250 nm): 1.93 min (minor), 2.17 min (major) (**Figure S4C**). Again, compound **7** was acylated in order to facilitate SFC analysis, which was performed on compound **8.** NMR spectra for (R)-terreazepine matched those of (S)-terreazepine.

To determine the stereochemical configuration of the natural terreazepine product, 1.3 mg of natural terreazepine was acylated as described above and subjected to SFC. Interestingly, natural terreazepine showed an enantiomeric ratio of 2:1 (S:R) (**Figure S4D**). To confirm that this was not due to the purification process itself, synthesized material was put through the same extraction and chromatographic separations as the natural compound, with an aliquot taken out at each step. Each aliquot was acylated and subjected to SFC (data not shown). No changes were witnessed in the enantiomeric ratios of synthetic compounds taken through this process, illustrating that the natural 2:1 ratio was not influenced by the chemical workup.

**References**

1. Clevenger, K. D.; Bok, J. W.; Ye, R.; Miley, G. P.; Verdan, M. H.; Velk, T.; Chen, C.; Yang, K.; Robey, M. T.; Gao, P., A scalable platform to identify fungal secondary metabolites and their gene clusters. *Nature chemical biology* **2017,** *13* (8), 895.

2. Clevenger, K. D.; Ye, R.; Bok, J. W.; Thomas, P. M.; Islam, M. N.; Miley, G. P.; Robey, M. T.; Chen, C.; Yang, K.; Swyers, M., Interrogation of benzomalvin biosynthesis using fungal artificial chromosomes with metabolomic scoring (FAC-MS): discovery of a benzodiazepine synthase activity. *Biochemistry* **2018,** *57* (23), 3237-3243.
